# Research on geometric parameter quantification of rail rolling contact fatigue crack damage based on 2D optical image

**DOI:** 10.1038/s41598-026-36276-w

**Published:** 2026-01-19

**Authors:** Yu Wang, Bingrong Miao, Ying Zhang, Zhong Huang

**Affiliations:** State Key Laboratory of Rail Transit Vehicle System, Southwest Jiaotong University, Chengdu, China

**Keywords:** Rolling contact fatigue crack, High-speed railway, Poisson reconstruction degree, Eddy current pulsed thermography (ECPT) imaging fusion, Engineering, Materials science

## Abstract

High-speed railway is a comprehensive carrier of high-technology. As an important infrastructure of high - speed rail tracks, rail breakage and derailment accidents caused by rail rolling contact fatigue crack damage are quite common. Considering that a single eddy current pulsed thermography cannot directly and very accurately make a quantitative estimate of the geometric parameters of Rolling Contact Fatigue (RCF), this paper proposes a rail contact fatigue crack damage identification system and method based on the fusion of 2D optical images and eddy current thermography. A mathematical - physical model of Poisson reconstruction degree for the fusion of 2D optical images and eddy current thermography is proposed to quantitatively characterize the geometric parameters of rail cracks, such as length and depth. A rail contact fatigue crack damage identification robot system based on the fusion of 2D optical images and eddy current thermography is designed and built. Experiments are carried out to compare the detection accuracy of crack geometric parameter identification in static and dynamic (different motion states) modes, as well as to study the impact on the quantification of crack geometric parameters. The experimental results show that the proposed Poisson reconstruction degree based on the fusion of 2D optical images and eddy current thermography has good robustness in quantifying crack geometric parameters in the slow - speed mode, verifying the effectiveness of the system and method.

## Introduction

**Fig. 1:**
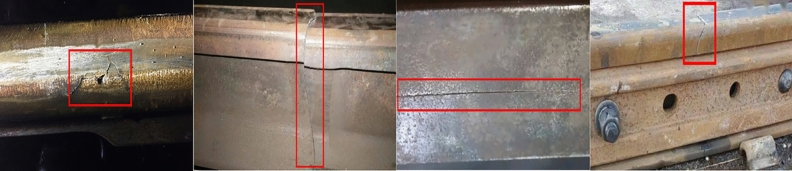
Rail crack damage.

Railway, as an important means of transportation, has embraced booming development opportunities. The rolling contact fatigue cracks (RCF) of rails are a general term for rail fatigue defects. The multi -crack phenomenon caused by material fatigue includes spalling cracks on the rail surface and in the shallow layer area, flat scars, and rail head cracks^1^. RCF in railway rails is a common safety hazard, attracting much attention. In the high - speed rail sector, due to the lack of sufficient inspection time, it has become a major hidden danger to railway safety. Figure 1 shows common surface crack damage of rails. In heavy - haul railways, it directly increases the amount of rail maintenance and replacement, while in the high-speed passenger transportation field, due to insufficient inspection time, it becomes a significant hidden danger to railway safety. Derailment and rail breakage accidents caused by RCF in rails are quite common^2^.

In the identification and detection of rail rolling contact fatigue crack damage, relevant scholars have conducted a large number of studies and experiments. Research on rail flaw detection methods can be traced back to 1877^3^. Since 1920, Dr. Sperry in the United States developed the Sperry inspection vehicle and put it into use^4^, making rail flaw detection an important periodic task. Over the past 100 years, rail detection technologies and equipment have been continuously improved and refined. From the early magnetic excitation methods such as magnetic flux leakage detection (MFL) to the ultrasonic detection method that gradually became dominant in the 1950s^5^, no less than 10 non - destructive testing (NDT) methods have been applied to rail defect detection. By using methods such as the time-delay focusing rule, it realizes large - area, visual ultrasonic detection technology^6^. Portable phased - array ultrasonic flaw detection equipment is used for manual detection of rail welds and fault points. However, due to factors such as speed, the rail inspection vehicle equipment based on phased - array ultrasonic technology is still in the research and development stage. TTCI from North America plans to integrate phased - array flaw detection technology into the rail dynamic flaw detection system^7^. The French company Socomate uses the Fast Array of Acoustic Signals Technology (FAAST)^8^, which has the advantage of exciting multiple ultrasonic beams in a single shot and intends to apply it to track detection to achieve efficient coverage of the rail head area. For RCF cracks, domestic scholars such as Lu Chao^9^ and Li Lianxiu used the time - of - flight diffraction (TOFD) technique to measure RCF defects in rails. Under static conditions, although TOFD technology can identify and detect the rail surface cracks to a certain extent, it is unable to identify and detect the rail surface cracks and other damages in a series of quantitative ways. At the same time, when the rail surface cracks are less than 4 mm, it is impossible to identify and detect, so TOFD technology has limitations in the identification and detection of steel surface damage.They attempted to detect RCF and made an attempt at flaw detection of internal flaws and inclined cracks using B - scan views. However, ultrasonic flaw detection technology is insufficient for detecting defects smaller than 4mm^10^.Electromagnetic non - destructive testing (EM NDT) methods have good detection capabilities for the metal surface and near - surface, and are used for rail surface flaw detection as a supplement to ultrasonic flaw detection^11^. Hall sensors are used to capture the leaked magnetic field signals, and it has good detection sensitivity for open defects on the rail head surface. However, as the speed increases, the eddy current excitation leads to a decrease in the magnetic flux leakage intensity and a decline in sensitivity. The pulsed eddy current (PEC) detection method is used for high - speed detection and can reach 75 km/h^12^. It has high detection sensitivity for surface and near - surface defects, but is affected by the lift - off effect^13^. The alternating current field measurement (ACFM) method can greatly overcome the lift-off effect, is suitable for RCF detection on the rail surface, and has an advantage in the quantitative detection of crack depth^14^. However, it is not very sensitive to subsurface non-open cracks, and there are mutual interference problems in the detection of multiple cracks. The electromagnetic acoustic transducer (EMAT) method is suitable for high - speed detection. It has high sensitivity to surface cracks between the probe emission and reception pairs, but also has lift-off limitations^15^. The "high-speed laser and video system" is currently widely used in various track detection systems, mainly for rapid positioning and evaluation of surface defects. Some experts predict that it is ineffective for rail contact fatigue cracks. However, several companies in Italy, the UK, and Germany have launched relevant track inspection vehicle equipment. An Italian company claims that it can identify cracks between 0.15mm- 0.5mm and provide their angles, lengths, and frequencies^16^. Alexander Machikhin et al.^17^ proposed a solution combining acoustic emission (AE) technology with digital image correlation (DIC) technology is proposed to address the problem of difficulty in accurately tracking and quantifying early fatigue cracks in steel rails. In the study, the initiation and propagation process of fatigue cracks in steel rails were simulated by cyclic loading, and the crack images were accurately geometrically quantified using DIC technology to continuously track the image evolution characteristics of cracks at different stages. At the same time, by calculating the maximum normal strain in the crack area and combining it with the strain threshold, the crack initiation time is determined, and the complete image time series data of the crack from appearance to propagation is recorded. David H. Allend et al.^18^ developed a bidirectional coupled multiscale computational model for predicting rail crack propagation under long-term cyclic loading, and validated the model using accumulated indoor cyclic crack propagation experimental image data from the past decade. By fixing the steel rail sample with a dedicated device and continuously capturing images of the entire process of internal defects evolving into cracks in the rail head, image sequences of crack length and shape changes under different cyclic loading times are obtained. The experimental images in the study are not only used to verify the accuracy of the model, but also the time evolution image sequence of cracks formed by itself can be used as a basis for distinguishing the possibility of different types of crack propagation.

Eddy current pulsed thermography is an electromagnetic induction thermography method. For conventional electromagnetic induction thermography, according to the time characteristics of the excitation signal, it can be divided into modulated eddy current thermography (ECLT)^19^ and eddy current pulsed thermography (ECPT)^20^.It can detect and evaluate defects during the heating and cooling stages of the material, thus having advantages such as high heating efficiency and fast detection speed. Abroad, the research on electromagnetic - excited thermography mainly focuses on eddy current pulsed thermography technology, with research institutions including the Fraunhofer Institute for Nondestructive Testing (IZFP) in Germany^21^, MTU Aero Engines in Germany^22^, Leoben University in Austria^23^, Laval University in Canada^24^, Newcastle University in the UK^25^, and the University of Bath in the UK^26^. Since the introduction of the eddy current pulsed thermography method, it has been widely used in the detection of metal materials, such as the detection of surface fatigue cracks in metal materials, stress evaluation of metal materials, rust detection under sprayed paint^27^, fatigue evaluation of key components such as gears and engine blade roots^28^. In China, the research on induction - excited thermography started relatively late, but good achievements have still been made. In 2003, with the support of the National 863 High - Technology Development Program, the Beijing Institute of Aeronautical Materials, Capital Normal University, etc. jointly established an infrared thermography detection laboratory, promoting the research and application of this non - destructive testing technology in China^29,30^. Other domestic universities, such as Nanjing University of Aeronautics and Astronautics, Sichuan University^31,32^, Shanghai Jiao Tong University, Xi’an Jiaotong University, National University of Defense Technology^33,34^, and the University of Electronic Science and Technology of China^34^, have successively launched research on the eddy current pulsed thermography method. They use FEM (Finite Element Method)to establish models, conduct post - processing of image data, and separate the spatial - temporal thermal characteristics existing in the thermal image sequence, conducting a large amount of research for the automatic detection, location, and quantification of defects.One of the core features of CT (computed tomography) measurement device is the form of energy input. The type and energy range of its energy source directly determine the detection principle, applicable scene and performance characteristics of the device. According to the difference of core energy input, CT measurement devices can be divided into the following main types, including industrial CT detection devices based on the characteristics of X-ray energy input, whose core energy input is X-ray, and the energy range is 10kev-1mev. Industrial CT detection device based on the characteristics of γ - ray energy input, the core energy input is γ - ray, and the energy range is 10kev-99mev. The industrial CE detection device based on the characteristics of neutron ray energy input, the core energy input is neutron beam, and the energy range is 1ev-1mev. The industrial CE detection device based on the characteristics of proton ray energy input, the core energy input is proton beam, and the energy range is 10mev-999mev. Due to the high requirement of core energy input and high cost based on γ - ray, neutron beam and proton beam, it is rarely used in the detection of industrial products.

The research on ECPT mainly includes four commonly used methods:(1) Method based on model determination.The disturbance mechanism of defects to eddy currents, establishment of defect models, study of the relationship between eddy current excitation and defects, and characteristics such as temperature distribution. This involves exploring how defects affect the eddy current field, creating mathematical or physical models to represent different types of defects, and analyzing how the eddy current excitation influences the defect and the resulting temperature distribution around it.(2)Method based on heat conduction analysis.The heat conduction mechanism and analysis model, which is used for the detection of deep - seated defects. This aspect focuses on understanding the principles of heat transfer within the material, developing theoretical models to analyze how heat diffuses in the presence of defects, and using these models to detect and locate defects that are deeper inside the material.(3)Method based on infrared thermal imaging.Enhancement and feature extraction of infrared thermal image sequences. Infrared thermal image sequences contain temporal characteristics of temperature, spatial characteristics of temperature, as well as the processes of eddy current action and heat diffusion, thus carrying abundant information. The goal is to extract useful features from these images and classify different types of defects based on these features.(4)Method based on feature quantification analysis.The problem of defect quantification. Based on the defect action mechanism and defect characteristics, relevant models are established to quantify the geometric information of defects, providing a basis for further defect evaluation. This means using the understanding of how defects interact with the eddy current and heat processes to create models that can determine the size, shape, and other geometric parameters of the defects, which is crucial for accurately assessing the severity and potential impact of the defects.

The quantitative analysis of rail damage mentioned above still has certain limitations, including the analysis of geometric quantification indicators such as length, width, depth, and angle of rolling contact fatigue cracks. For natural defects, such as RCF crack defects in steel rails, the defects extend inward from the surface at a certain inclination angle, which differs from the thermal phenomenon of vertical cracks. If the angle crack model is used to study the asymmetric distribution of heat through simulation, there will inevitably be certain limitations. Surface cracks have a certain depth, and the research on the mixing problem of Joule heat and thermal diffusion caused by eddy current disturbance during the detection process is not sufficient, and there are still significant problems in depth quantification. At the same time, there are often impurities in the gaps of natural cracks, and traditional damage detection methods also have certain limitations. Therefore, the study of quantifying surface damage on rails is particularly important and has great scientific research significance. A multidimensional comparative analysis was conducted on commonly used non-destructive testing methods such as ultrasonic testing, eddy current testing, magnetic particle testing, and visual testing. The specific advantages and disadvantages of the comparative results are shown in Table 1. Quantitative rating table (out of 10 points, the higher the score, the better the performance).

**Table 1:** Comparison results of advantages and disadvantages.

Method	Detection accuracy	Detection cost	Detection adaptability	Detection depth	Detection speed	Automation level (low=high score)
Ultrasonic testing	9	4	6	9	5	5
Magnetic particle Testing	8	7	7	3	7	6
Eddy current testing	7	6	8	4	9	8
Visual inspection	6	9	5	2	8	9

The remaining content of this paper is as follows. In Section “Methodology”, the specific methods are described, and the basic theory of ECPT is analyzed. The mathematical model of the physical relationship between crack geometric parameters and the Poisson reconstruction degree of imaging fusion is also presented. In Section “Experiment set-up”, the relevant experimental settings, the construction of the experimental environment, and the experimental process are introduced in detail. In Section “Results and analysis”, a comparative analysis of the previous experimental results is carried out and discussed. In Section “Conclusion and future work”, conclusions are drawn, and the plans for the next - step research are made.

## Methodology

### 2D optical imaging processing flow

**Fig. 2:**
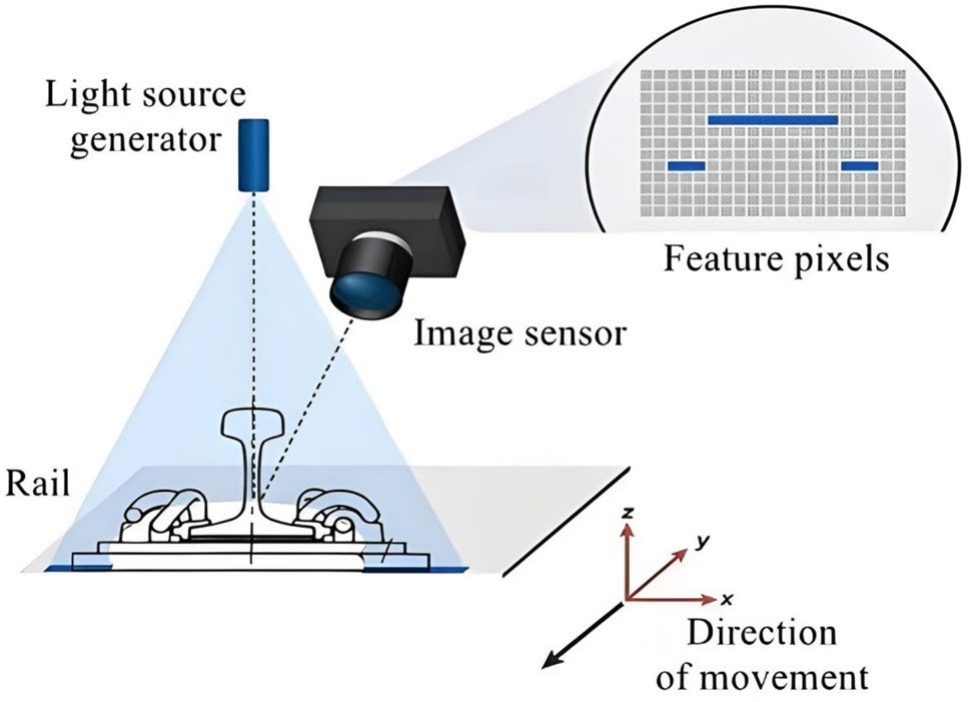
Schematic diagram of 2D optical imaging principle for rails.

Machine vision technology based on 2D image sensors acquires rail images. Then, in combination with developed image - processing algorithms, it conducts a series of operations on these rail images. These operations include grayscale transformation, feature enhancement, feature morphological operations, feature extraction, feature location, and recognition. Through these steps, the initial location and recognition of rail crack damage can be achieved. Figure 2 shows the schematic diagram of 2D optical imaging of the rail.

For the 2D rail imaging system to accurately conduct the initial location and identification of cracks, it mainly depends on whether high - quality 2D rail images can be collected. The collection of a high - quality 2D rail image is determined by the optical characteristics of the rail surface. The optical elements that affect the imaging effect of the rail mainly include the reflected light of the incident light, the transmitted light that penetrates into the interior of the rail, and the radiation light emitted by the object itself.

In the actual 2D image acquisition of steel rails, appropriate filters can be added according to the actual environment and imaging requirements to eliminate the influence of self radiation light.

**Fig. 3:**
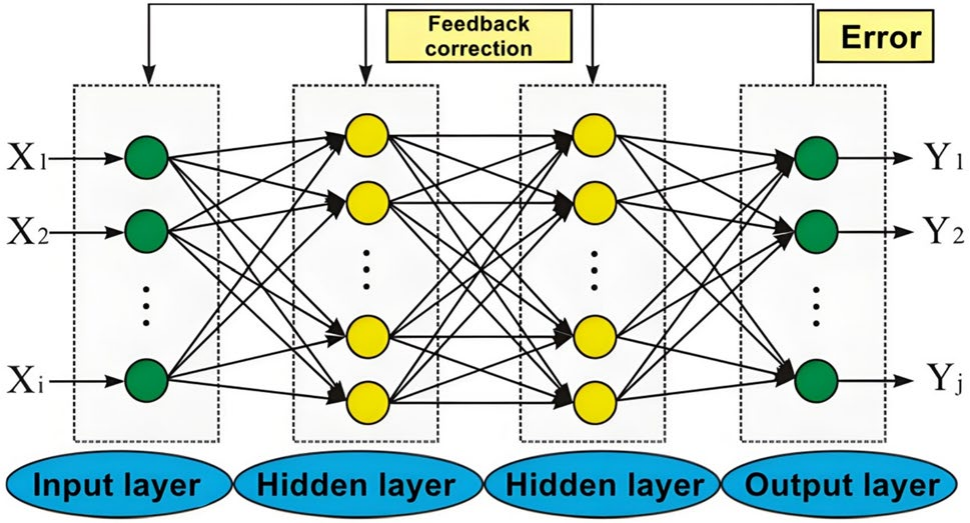
BP neural network structure diagram.

In the process of processing crack features in 2D images of steel rails, the improved BP neural network algorithm is mainly used to achieve initial localization and recognition of surface cracks on the rail. The improved BP neural network algorithm model mainly includes four layers. Firstly, the 2D image of the rail surface crack is collected, and the extracted color features, texture features, shape features and other feature parameters from the rail surface image are fused. The fused feature parameters are used as inputs to the network model. Increasing the number of hidden neurons can correspondingly improve the training accuracy of the neural network. As shown in Figure 3 is the BP neural network structure diagram, which mainly consists of an input layer, a hidden layer, and an output layer. The allowable range of expected error should comprehensively consider various factors, and the output error E should be fed back to the input layer and hidden layer to correct the entire process of the neural network and further reduce system errors. The specific expected error is finally obtained through sample training.

The selection of its architecture mainly considers the characteristics of rail surface damage, including surface damage types, damage characteristics, etc., and the amount of rail damage characteristics data, as well as the actual operating conditions. The output layer includes a two category task and a multi category task. The two category task is divided into rapid response identification of rail surface damage and no damage. The number of nodes in the output layer is equal to 1. The multi classification task is divided into different types of rail surface damage, such as cracks, pits, wear, etc. the number of output layer nodes and the number of damage categories match. The input layer is mainly based on the matching degree of rail damage feature data. After preprocessing, the effective features of the damage feature are extracted, and then they are input into the network. The number of nodes in the input layer is matched with the number of feature dimensions extracted. The hidden layer takes the extracted rail surface damage characteristics as the core, and is divided into three layers of deep network based on rail surface damage type, damage characteristics and damage fusion. The sample size of rail damage in this paper is 5000 rail damage feature images. The number of samples for feature training, test verification and verification of damage features is 5000, mainly including rail surface crack damage, a small amount of wear damage, a few pit damage, and no damage. In the neural network, the input layer: 20 nodes, mainly to extract the key damage features of 20 dimensional rail surface. The hidden layer includes 112 nodes. At the same time, a batch normalization layer is added after the hidden layer to stabilize the input distribution, which is conducive to accelerating the training convergence. The final output layer is 4 nodes, which mainly matches the previous sample types.

Firstly, the collected rail damage feature images are subjected to denoising processing. By weighting the mean, the rail damage feature pixels are made more prominent. The denoising filtering process represents:

1 $$M^{\prime}(x,y) = \frac{1}{K}\sum {_{{\left( {x,y} \right) \in s}} \,} \cdot G\left( {i,j,\sigma } \right)\; \cdot \;M(x + i,y + j)$$

The denoising filter kernel is:

2 $$G(i,j,\sigma ) = \frac{1}{{2\pi \sigma^{2} }}e^{{ - \frac{{i^{2} + j^{2} }}{{2\sigma^{2} }}}}$$

Secondly, by constraining the number of nodes in the network layers, the robustness of the training model can be improved by avoiding excessive number of layers and neurons, limiting the absolute values or sum of squares of parameters, and preventing overfitting caused by excessively large parameters. The essence is to reduce complexity by limiting the total number of parameters in the network (number of neurons x connection weights), which can be formulated as constraints on the network structure and expressed as:

3 $$\sum\nolimits_{i = 1}^{N - 1} {\eta_{i} \cdot \le C;L \le D}$$

The number of neurons in layer $l$ of the network is $n_{l}$ in the input layer dimension, $n_{0}$ in the output layer dimension ($n_{l}$is the total number of layers), $L$ is the upper limit of the total number of neurons, and $C$ is the maximum number of layers in the network.

Finally, by combining the infrared thermal image of rail damage features with the 2D optical image of rail damage features through cross fusion and fusion of damage feature images, it can be represented as:

4 $$F_{{{\mathrm{fusion}}}} = W_{vis} \cdot F_{{{\mathrm{vis}}}} + W_{{{\mathrm{ir}}}} \cdot F_{{{\mathrm{ir}}}}$$

$W_{vis} ,W_{ir}$ are the fusion weight matrices obtained by training the model.

Through the following experimental test results, it shows good performance, especially in the dynamic test process, the average absolute quantization error is 0.109%, and the mean square quantization error is about 0.018%, which is better than the static test results. It shows some advantages in the identification and detection of rail surface damage, and can be extended to the identification and detection of rail damage in urban rail transit, including metro rail.

For each 2D image sample of rail surface cracks collected, The activation function can be expressed as: $f(x) = \max (0,x_{n} )$, mainly for the problem of abnormal gradient descent during the operation process, mainly concentrated in several hidden layers. The improved algorithm sets multiple hidden layer neurons, and if the number of neurons exceeds the set limit, the hidden layer fitting will further concentrate the damage feature data of the sample. Use the collected 2D feature images of surface damage cracks on the steel rails as the actual input features of the neural network, and use the extracted steel rail crack feature data as the output. assuming that the input of the element in n iterations is represented as $x_{i} (n)$, then the input of element j can be represented as $p_{j} (n)$:

5 $$p_{j} (n) = \sum\limits_{i = 0}^{n} {w_{ij} } (n)x_{i} (n)$$

In formula (7), *N* represents the number of units in input $j$, $w_{ij}$ represents the weight function between units, and assuming $f_{j} \left( p \right)$ is its action function, the output of unit j is expressed as:

6 $$y_{j} (n) = f_{j} (P_{j} (n))$$

Assuming the expected output is $d_{j} (n)$ and the error signal is $E_{j} (p)$, the total squared error of the output can be expressed as:

7 $$\xi (n) = \frac{1}{2} \cdot \sum\limits_{j = 1}^{N} {E_{j}^{2} } (n)$$

In formula (9), M is the number of output terminals, and the total number of training sets is N. Therefore, the mean square error can be expressed as:

8 $$\overline{\xi (n)} = \frac{1}{N} \cdot \sum\limits_{n = 1}^{N} \xi (n)\xi (n)$$

The compensation value of the weight function can be expressed as:

9 $$\begin{array}{*{20}l} {\Delta w_{ij} (n)} \hfill & { = - \eta \frac{\vartheta \xi (n)}{{\vartheta w_{j} }}} \hfill \\ {} \hfill & { = - \eta \delta_{j} (n)x_{i} (n)} \hfill \\ \end{array}$$

The negative sign in formula (11) represents the correction amount in the direction of gradient descent, and $\delta_{j} (n)$ is the gradient function. The weight can be expressed as:

10 $$w_{ij} (n + 1) = w_{ij} (n) + \eta \delta_{j} (n)x_{i} (n)$$

On this basis, the momentum factor and 2D image active learning samples of rail cracks were added, and the improved weights can be expressed as:

11 $$\Delta w_{j} (n + 1) = \eta \delta_{j} x_{j} (n) + m\Delta w_{j} (n)$$

In formula (13), mc is the momentum factor, with values ranging from 0.01 to 1.00. The improved learning rate is represented as:

12 $$\eta_{(t).} = \eta_{(t - 1)} \left( {1 - \frac{l}{T + C}} \right)$$

In formula (14), t and T are the number of iterations and the total number of iterations, respectively, and C is an integer greater than 0.

### Eddy current thermal imaging processing flow

**Fig. 4:**
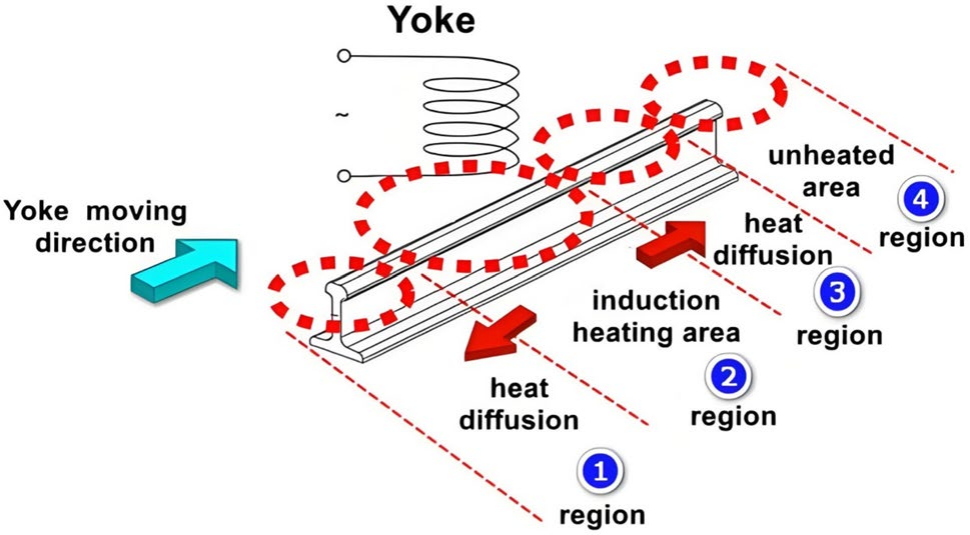
Schematic diagram of eddy current thermal imaging rail crack detection method.

After conducting initial positioning and identification of surface cracks on steel rails using 2D optical imaging, the crack area that can be located and identified through eddy current thermal imaging will be larger, encompassing both the crack area on the steel rail surface and the normal area. Eddy current is induced on the material surface and interior through the excitation coil, generating Joule heat. Heat is conducted on the surface and inside of materials, and surface or subsurface defects of materials have an impact on heat conduction, ultimately reflected in changes in the temperature field distribution on the material surface. Figure 4 illustrates the schematic diagram of the eddy current thermal imaging method for detecting rail cracks. Among them, region ① is the heat conduction region, region ② is the induction heating region, region ③ is the heat conduction region, and region ④ is the unheated region. The temperature in region ④ is the lowest (close to ambient temperature) because it is far away from the heat source and is not affected by thermal conduction. Due to the thermal diffusion of the heat source, the temperature in region ③ gradually increases from right to left. The temperature in region ③ rises sharply due to the direct heating by eddy currents. Notably, the temperature drop in region ① is attributed to an increase in distance from the heating coil, despite it having been previously heated.

When the heating coil is subjected to alternating excitation with a frequency of f, according to the law of electromagnetic induction, an induced eddy current of the same frequency will be generated. As described by Maxwell’s equations, the differential equation for the alternating electromagnetic field can be expressed as follows:

13 $$\left\{ {\begin{array}{*{20}l} {\nabla \times \overline{H} = \overline{J} + \frac{{\vartheta \overline{D}}}{\vartheta t}} \hfill \\ {\nabla \times \overline{E} = - \frac{{\vartheta \overline{B}}}{\vartheta t}} \hfill \\ {\nabla \cdot \overline{D} = \rho } \hfill \\ {\nabla \cdot \overline{B} = 0} \hfill \\ \end{array} } \right.$$

In formula (15), $\overline{H}$ and $\overline{B}$ denote magnetic field strength and magnetic flux, respectively, $\overline{E}$ represents electric field strength, $\overline{j}$ represents current density flowing through the coil, $\overline{D }$ represents electric displacement vector, $\rho$ represents charge density, and $t$ represents time. During the eddy current thermal imaging detection process, the eddy current frequency in the heating coil typically ranges from tens of KHz to MHz. Hence, the electromagnetic field mathematical equation for eddy current thermal imaging, based on Maxwell’s equations, can be expressed as follows:

14 $$\sigma \frac{{\vartheta \overline{A}}}{\vartheta t} + \nabla \times \left\{ {\frac{1}{\mu }\nabla \times \overline{A}} \right\} - \sigma \overline{v} \times \left( {\nabla \times \overline{A}} \right) = \overline{J}_{s}$$

In formula (16), $\mu$ represents the magnetic permeability of the material, $\sigma$ represents the electrical conductivity, and $\overline{J}_{s}$ represents the source current density.

**Fig. 5:**
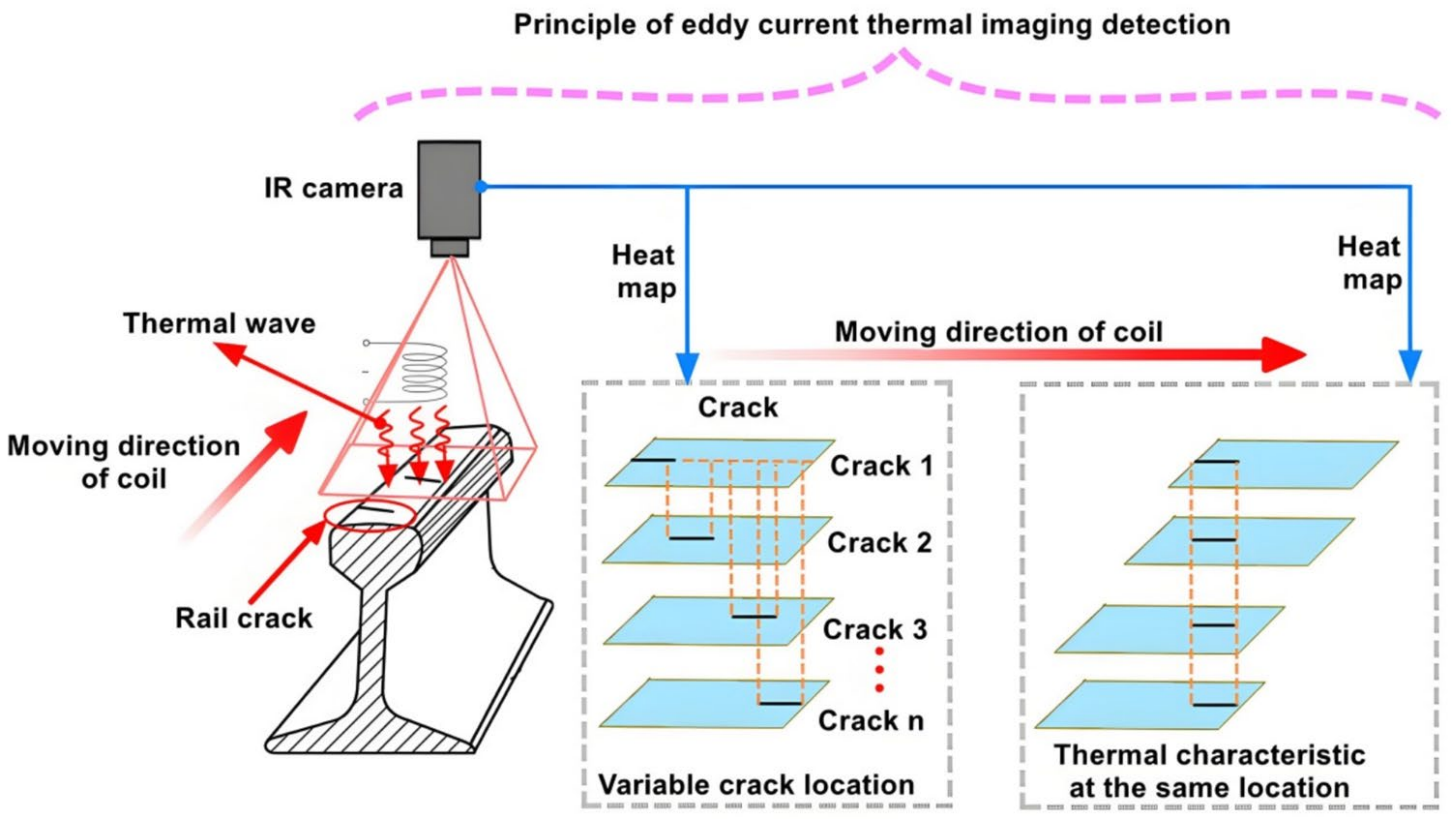
Schematic diagram of eddy current thermal imaging detection.

Based on the preceding analysis, it is evident that temperature varies with time and space, and distinct temperatures are observed at the surface cracks of the rail. Since the eddy current coil detects rail surface cracks in motion, the temperature at these cracks continuously changes with the coil’s movement. Hence, it is imperative to conduct feature fusion analysis on the 2D image and eddy current thermal imaging of the rail surface cracks. Following initial positioning and identification, quantitative analysis of the geometric dimensions of the rail surface cracks can be performed. Figure 5 illustrates the schematic diagram of eddy current thermal imaging detection in motion.

### Proposed a new fusion method based on Poisson reconstruction degree

After acquiring 2D optical images of rail surface cracks and deriving temperature thermal characteristic maps of these cracks using eddy current thermal imaging, a novel fusion approach was employed. This method integrates the 2D optical images of rail cracks with the thermal characteristic maps obtained from eddy current imaging, based on Poisson reconstruction degree. The aim is to precisely locate and identify rail surface cracks, while acquiring detailed geometric quantification parameters of these cracks. Initially, it is crucial to compute the gradient field between the 2D optical image of the crack and the thermal characteristic map derived from eddy current imaging. The formula for calculating this gradient field is as follows:

15 $$\begin{gathered} \nabla^{2} \varphi = \frac{{\vartheta^{2} \varphi }}{{\vartheta x^{2} }} + \frac{{\vartheta^{2} \varphi }}{{\vartheta y^{2} }} \hfill \\ grad\varphi = \nabla \varphi \hfill \\ \end{gathered}$$

For the crack thermal characteristic map images of 2D optical images and eddy current thermal imaging, assuming the corresponding image functions are:

16 $$G = f(x,y)$$

The corresponding divergence can be expressed as:

17 $$\nabla \cdot \nabla \varphi = div(grad\varphi )$$

18 $$\Delta \varphi = G\left( f \right)$$

Next, we can directly solve and obtain the Poisson equation under fusion, which can be expressed as:

19 $$H_{x} = b$$

In formula (21), $H$ is the coefficient matrix, $x$ is the initial value of crack feature pixels under fusion, $b$ is the divergence obtained earlier, where coefficient matrix $H$is non singular and $a_{ii} \ne 0\;(i = 1,2, \ldots ,n)$, then coefficient matrix $H$ can be expressed as:

20 $$H = \left[ {\begin{array}{*{20}c} 0 & 0 & \ldots & 0 \\ {a_{21} } & 0 & \ldots & 0 \\ \ldots & \ldots & \ldots & \ldots \\ {a_{n1} } & {a_{n2} } & \ldots & 0 \\ \end{array} } \right] + \left[ {\begin{array}{*{20}c} 0 & {a_{12} } & \ldots & {a_{1n} } \\ 0 & 0 & \ldots & {a_{2n} } \\ \ldots & \ldots & \ldots & \ldots \\ 0 & 0 & \ldots & 0 \\ \end{array} } \right]$$

Define the set of 2D optical image crack feature pixels for rail surface cracks as $P$, which can be represented as $P\,(m,n)$; the set of feature pixels for eddy current thermal imaging crack images as $Q$, which can be represented as $Q\left( {m,\;n} \right)$; the threshold for crack feature images is represented as $R$, the threshold for image foreground features is represented as $T$, and the threshold for feature and background segmentation is represented as $K$. Therefore, there are de contextualization representation methods:

21 $$P(m,n) = \left\{ \begin{gathered} 1{\kern 1pt} {\kern 1pt} {\kern 1pt} {\kern 1pt} {\kern 1pt} {\kern 1pt} {\kern 1pt} {\kern 1pt} {\kern 1pt} {\kern 1pt} {\kern 1pt} {\kern 1pt} {\kern 1pt} {\kern 1pt} T \in (K,R) \hfill \\ 0{\kern 1pt} {\kern 1pt} {\kern 1pt} {\kern 1pt} {\kern 1pt} {\kern 1pt} {\kern 1pt} {\kern 1pt} {\kern 1pt} {\kern 1pt} {\kern 1pt} T \in (0,K) \hfill \\ \end{gathered} \right.P(m,n) = \left\{ \begin{gathered} 1{\kern 1pt} {\kern 1pt} {\kern 1pt} {\kern 1pt} {\kern 1pt} {\kern 1pt} {\kern 1pt} {\kern 1pt} {\kern 1pt} {\kern 1pt} {\kern 1pt} {\kern 1pt} {\kern 1pt} {\kern 1pt} T \in (K,R) \hfill \\ 0{\kern 1pt} {\kern 1pt} {\kern 1pt} {\kern 1pt} {\kern 1pt} {\kern 1pt} {\kern 1pt} {\kern 1pt} {\kern 1pt} {\kern 1pt} {\kern 1pt} T \in (0,K) \hfill \\ \end{gathered} \right.$$

If the crack feature pixels of 2D optical images that can be retrieved are $P1,\;P2,\;P3, \ldots ,Px$, $P1\left( {m1,n1} \right)$, $P(m2,n2)$, ……, $Px(mx,\;nx)$ then the statistical result of feature pixels is $S1$:

22 $$S1 = \sum\limits_{y = 1}^{t} \frac{1}{2} \cdot (P1 + P2)$$

If the crack feature pixels of eddy current thermal imaging that can be retrieved are $Q_{1} ,Q_{2} ,Q_{3} , \ldots ,Q_{r}$, $Q1\left( {m1,n1} \right)$, $Q2(xm^{2} ,n2)$,……, $Qx\left( {mx,\,nx} \right)$, then the statistical result of feature pixels 6 is as follows::

23 $$S2 = \sum\limits_{y = 1}^{i} \frac{1}{2} \cdot (Q1 + Q2)$$

From this, the Poisson reconstruction degree function equation under fusion can be expressed as:

24 $$\nabla \cdot M = \frac{{S{1}(p)}}{S2(q)}$$

Finally, the crack feature pixel $\overline{x}$ based on Poisson reconstruction degree under fusion can be obtained, which can be used for localization recognition and quantitative analysis of geometric parameters of the obtained crack feature pixels. Can be expressed as:

25 $$\overline{x} = \nabla M \cdot x$$

## Experiment set-up

### Rail crack detection system based on 2D optical image eddy current thermal imaging fusion

**Fig. 6:**
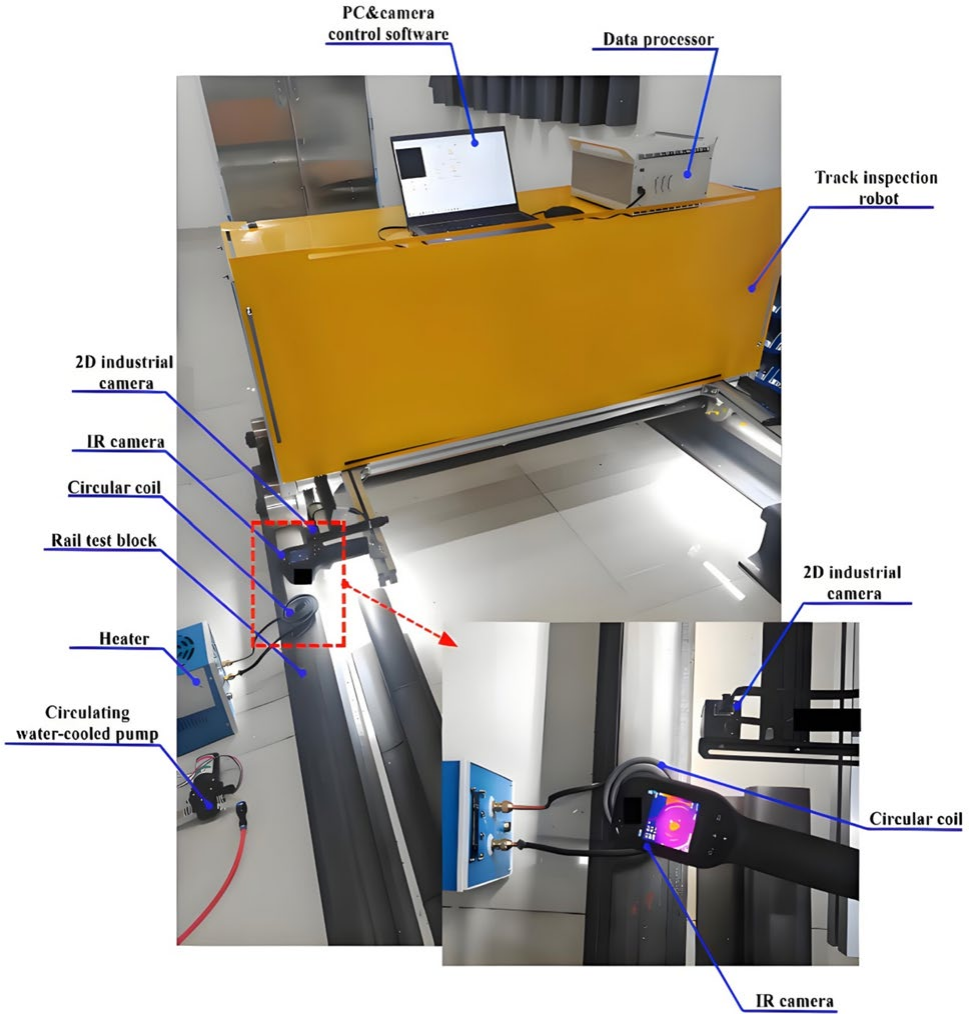
Field diagram of rail crack detection system.

Experiments and verifications were conducted under both dynamic and static conditions for different motion state modes, as illustrated in Figure 6, which depicts a constructed 2D optical image-eddy current thermal imaging fusion system for rail crack detection using a robot. The core components of this designed and developed rail crack detection robot system include an infrared camera, control software system, 2D optical industrial camera, electromagnetic induction heating module, and a water cooling device. The infrared camera used in this system is a HIKMICRO E09 model, featuring a sampling frequency of 50 Hz with a maximum capability of 100 Hz, and a measurement accuracy of ±2℃. The 2D optical industrial camera utilizes a 5-megapixel GIGE interface, boasts a frame rate of 30fps, and employs an eddy current heating power of 3kw. It maintains continuous heating during both low-speed and high-speed state detection, achieving a maximum detection speed of 2 m/s and maintaining heating for over 10 seconds. The coil adopts a circular disc-shaped design, positioned 5 mm away from the steel rail surface.

### Description of specimens

In this paper, artificial cracks on the rail surface rolled by train are used to simulate idealized RCF cracks. The steel grade of the rail is U75V, and its chemical composition is shown in Table 2.

**Table 2:** Chemical composition (%) of the rail steel.

C	Si	Mn	P	S	V	Al
0.75	0.62	0.93	0.015	0.007	0.05	0.002

**Fig. 7:**
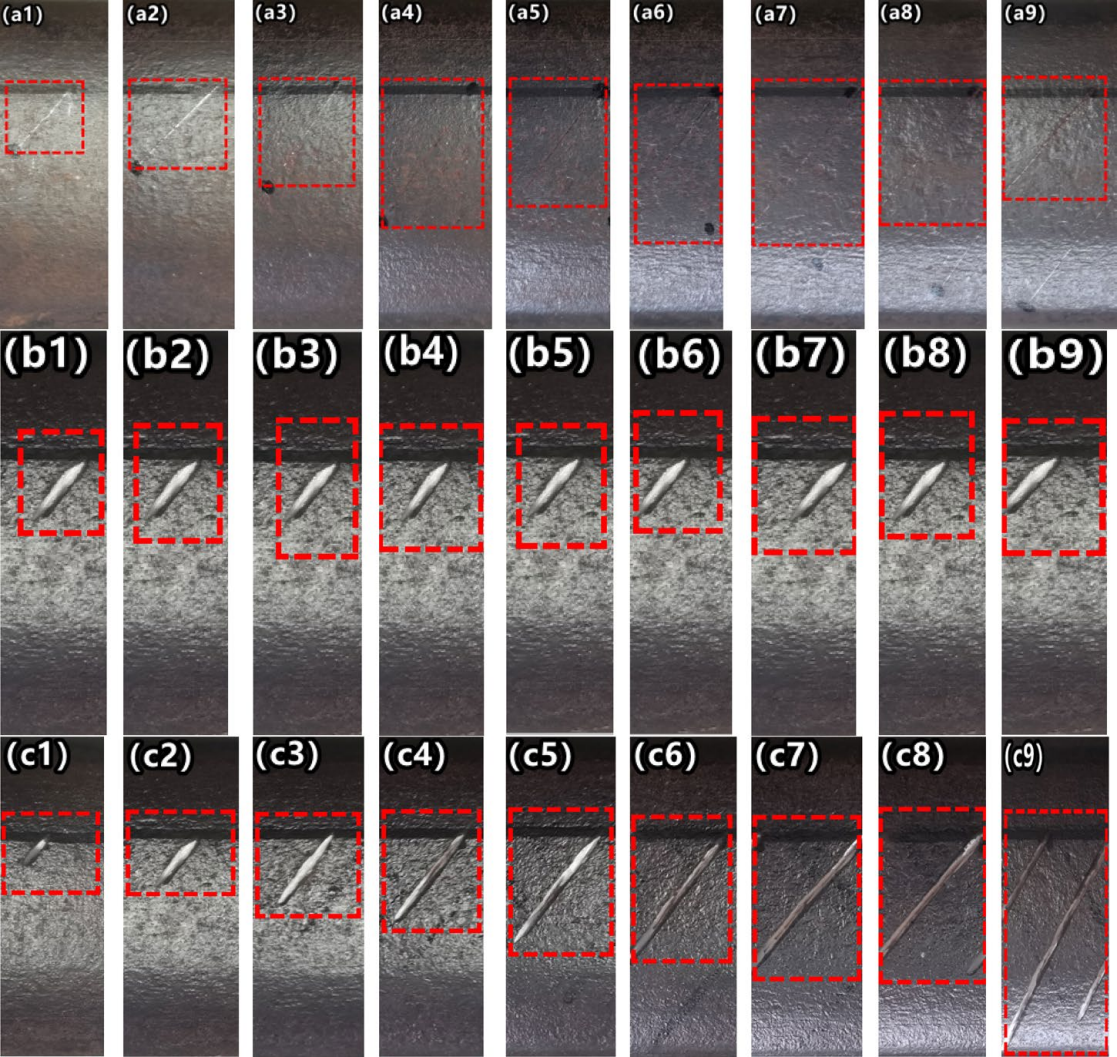
Crack images with different geometric parameters (length, depth, width).

The rail sample, with a total length of 6 meters, is welded from two 3-meter-long 60-gauge rails. The wire cutting technology is mainly used to create the required artificial cracks.Based on the operational status of high-speed trains, three distinct types of cracks are created at intervals on the rail samples. The first type comprises nine sets of cracks of varying lengths, the second type has nine sets of cracks of differing depths, and the third type features nine sets of cracks with varying widths. The spacing between cracks within each group is 200 mm, while the spacing between different types is 300mm. Multiple sets of experimental tests were conducted on these three distinct types of cracked rail samples. As illustrated in Figure 7, (a1-a9) represent cracks of different lengths in the first type, (b1-b9) depict cracks of varying widths in the second type, and (c1-c9) show cracks with differing depths in the third type. Considering the real and actual working conditions of the track rails on the railway line, as well as the surface crack damage of the rails on the line, the artificial manufacturing of cracks in the article is completed according to the standard "Railway Line Repair Rules" (Tieyun [2006] No. 146).

The first, second, and third types of cracks were selected as the objects of experimental analysis. Specifically, the first type had a depth of 2 mm, the second type had a length of 5 mm, and the third type had a width of 0.2mm. Additionally, the spatial angles (measured against the train’s running direction and the horizontal plane) were consistent for all three types of cracks. For the first and third types of cracks, the length ranged from 1 mm to 10 mm; for the second and third types, the depth varied from 0.35mm to 8.00mm; and for the first and second types, the width spanned from 0.1mm to 5.00mm. Detailed dimensions of the cracks can be found in Table 3.

**Table 3:** Detailed dimensions of cracks.

	Crack number	#1	#2	#3	#4	#5	#6	#7	#8	#9
1 st group	Lengths on the tread(mm)	1	2	3	4	5	6	7	8	10
	Width(mm)	0.1	0.2	0.3	0.4	0.6	0.8	1.0	1.5	2.0
	Crack depth(mm)	2	2	2	2	2	2	2	2	2
2nd group	Lengths on the tread(mm)	5	5	5	5	5	5	5	5	5
	Width(mm)	0.1	0.2	0.3	0.4	0.6	0.8	1.0	1.5	2.0
	Crack depth(mm)	0.35	0.5	1.0	1.5	2.0	2.5	3.5	5	8
3rd group	Lengths on the tread(mm)	1	2	3	4	5	6	7	8	10
	Width(mm)	0.2	0.2	0.2	0.2	0.2	0.2	0.2	0.2	0.2
	Crack depth(mm)	0.35	0.5	1.0	0.5	2.0	2.5	3.5	5	8

## Results and analysis

### Experimental results of cracks at different depths under different states

#### Experimental test results of different crack depths under static conditions

**Fig. 8:**
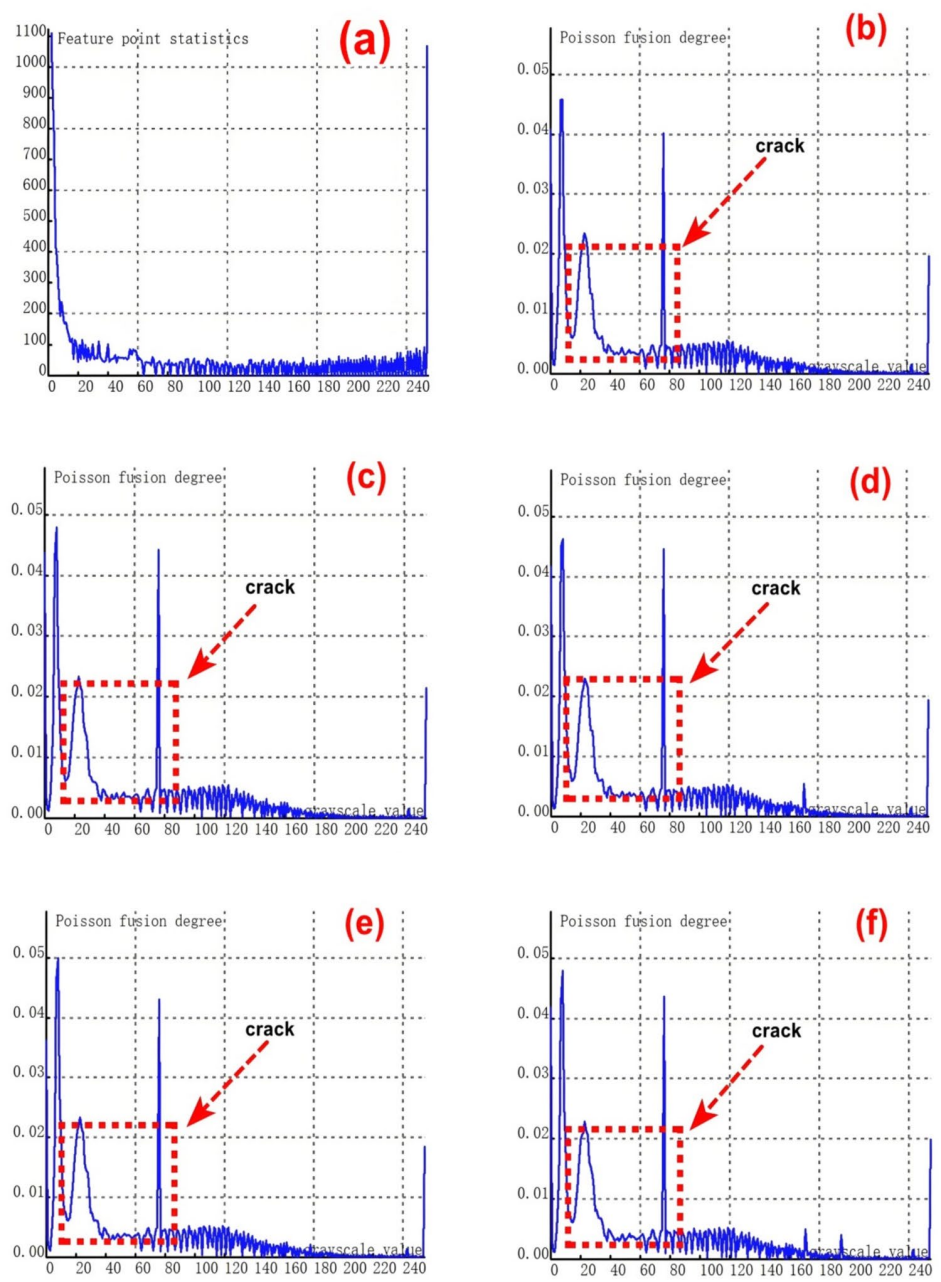

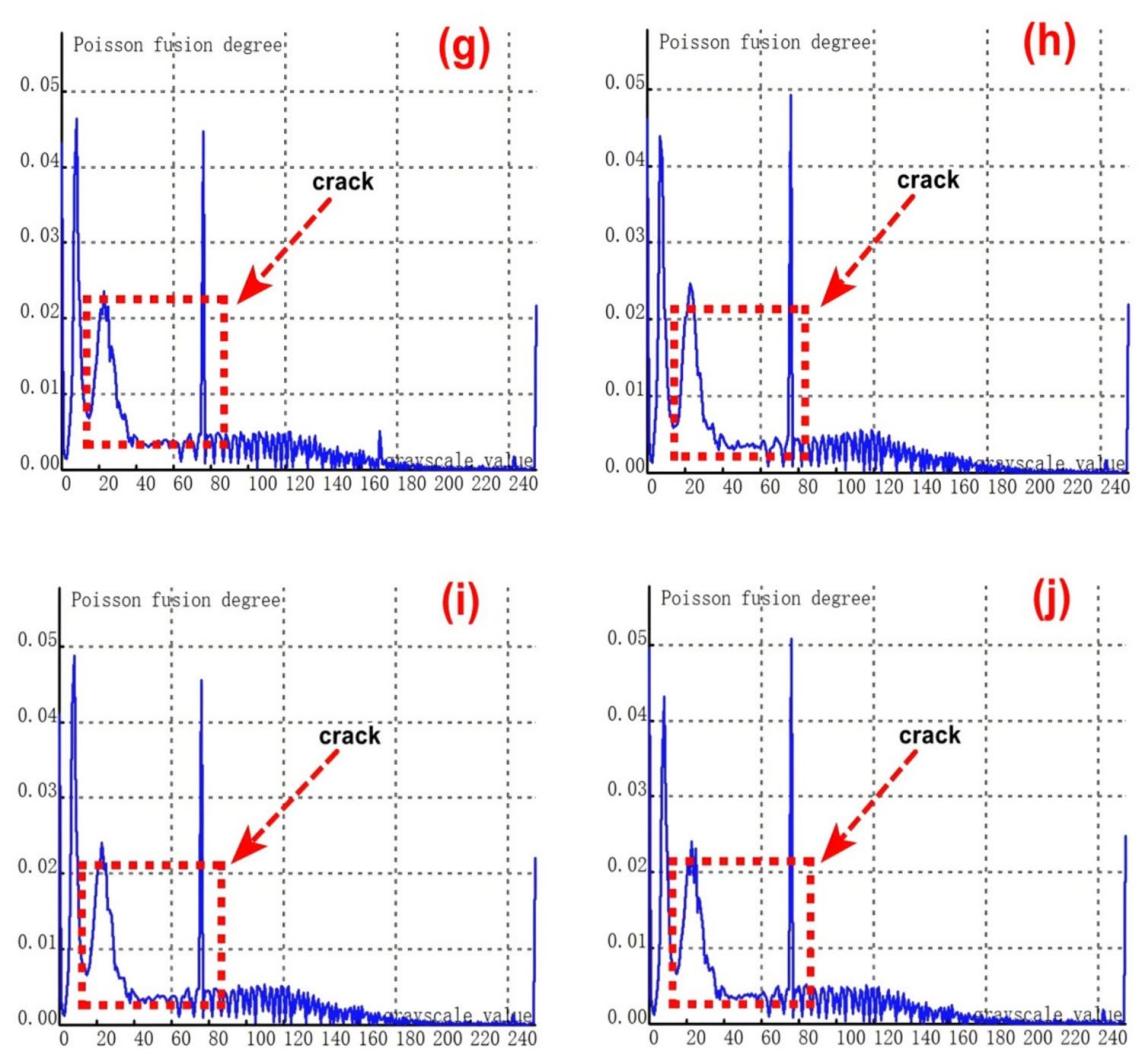
Poisson reconstruction degree relationship curve of cracks at different depths under static conditions.((**a**). Control group; (**c**). Analysis results of the second type # 2 test in Table 3; (**d**). Analysis results of the second type # 3 test in Table 3; (**e**). Analysis results of the second type # 4 test in Table 3; (**f**). Analysis results of the second type # 5 test in Table 3; (**g**). Analysis results of the second type # 6 test in Table 3; (**h**). Analysis results of the second type # 7 test in Table 3; (**i**). Analysis results of the second type # 8 test in Table 3; (**j**). Analysis results of the second type # 9 test in Table 3.).

To validate the stability and reliability of the novel method for feature - fusion detection of cracks at varying depths, which is based on Poisson reconstruction degree fusion, a second set of samples containing 9 groups of cracks with different depths was chosen for experimental testing. The crack depths range from 0.35 to 8.00 mm.As depicted in Figure 8(a), it shows the relationship curve between crack feature points and feature grayscale values without undergoing fusion processing. During the image acquisition, due to the presence of cracks, visible light fails to be directly reflected to the camera at the crack positions. Consequently, the grayscale values at the crack feature locations are relatively low, and the number of collected crack - feature pixels is limited. This situation poses significant challenges to crack recognition.From the figure, it is evident that crack feature points fall within two grayscale value ranges: 0 - 20 and 240 - 255. Additionally, there is a relatively large number of feature points within the intermediate grayscale range, which severely interferes with the identification of crack features.Subsequently, further fusion processing was carried out. The experimental results of crack testing under different Poisson reconstruction degrees are presented in Figures (b - j).

By examining Figures 8(b-j), it becomes apparent that the fused crack features are highly distinct. The Poisson reconstruction degree of the fused crack features reaches its peak within the grayscale value ranges of 10- 20 and 60–80. In other words, the Poisson fusion degree at both ends of the fused crack attains its maximum.The Poisson fusion degree in the middle feature region of the fused crack is relatively lower. This is because, after the fusion process, the crack features are more pronounced compared to the pre - fusion state. Specifically, the distinguishability of the crack edge features is significantly enhanced, which is primarily associated with the crack surface’s absorption and reflection of visible light.It is evident that for crack features of the same finite length, taking the aspect ratio into account can also exert a certain influence on the results. Consequently, in Figure 7, the maximum Poisson reconstruction degree occurs at different grayscale values.

The relationship between the crack depth and the Poisson fusion degree of crack feature images was analyzed through least - squares fitting. As presented in Table 4, there is an approximately monotonic and linear increase between the crack depth and the Poisson reconstruction degree. Consequently, the experimental data of crack depth were subjected to linear fitting. A calibration curve was then derived based on the parameters of the fitting curve to quantitatively assess the crack depth. The quantization results are displayed in Table 4 and Figure 8. The average absolute quantization error stands at 0.16%, and the quantization mean - square error is approximately 0.05%. These results indicate that the Poisson fusion degree is effective in characterizing the crack depth.

**Table 4:** Quantitative results of crack testing based on Poisson reconstruction degree.

Reference crack depth(mm)	0.35	0.5	1.0	1.5	2.0	2.5	3.5	5	8
Maximum Poisson reconstruction degree	0.046	0.048	0.047	0.05	0.048	0.047	0.049	0.049	0.052
Quantitative depth(mm)	0.32	0.52	0.92	1.56	2.1	2.6	3.41	4.2	7.8
Quantitative error (%)	−0.09	0.04	−0.09	0.04	0.05	0.04	−0.03	−0.19	−0.03
Mean Absolute Error	0.16								
Mean Squared Error	0.05								

#### Experimental test results of different crack depths under dynamic conditions

**Fig. 9:**
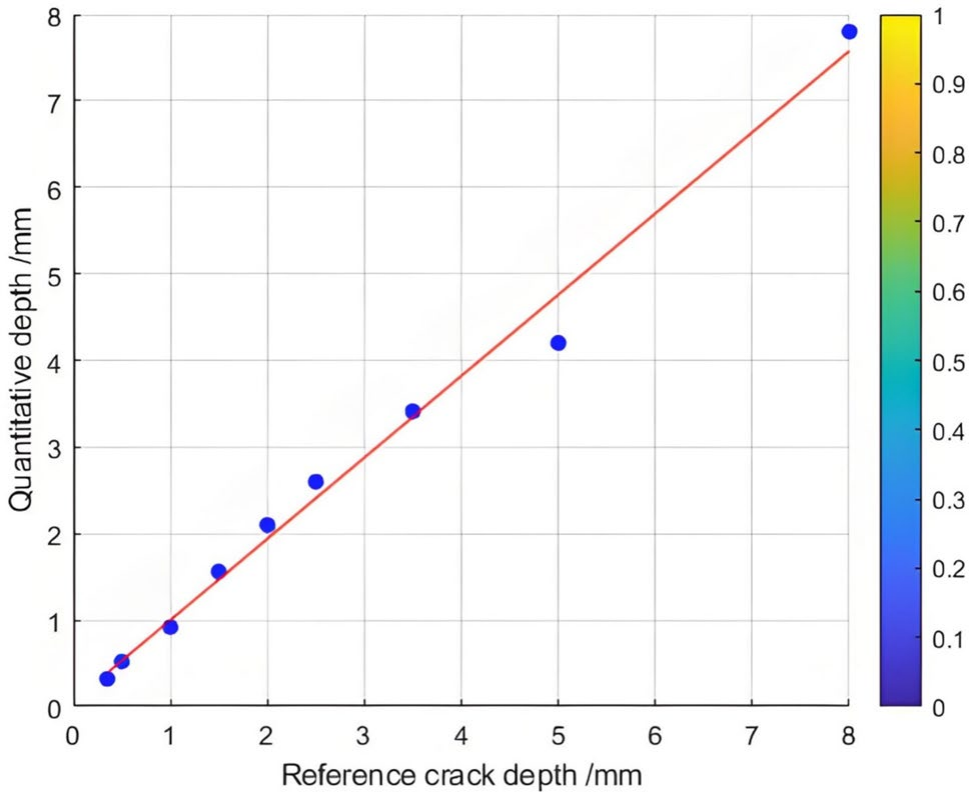
Poisson reconstruction degree relationship curve of cracks at different depths under dynamic conditions. ((**a**). Control group;; (**c**). Analysis results of the second type # 2 test in Table 3; (**d**). Analysis results of the second type # 3 test in Table 3; (**e**). Analysis results of the second type # 4 test in Table 3; (**f**). Analysis results of the second type # 5 test in Table 3; (**g**). Analysis results of the second type # 6 test in Table 3; (**h**). Analysis results of the second type # 7 test in Table 3; (**i**). Analysis results of the second type # 8 test in Table 3; (**j**). Analysis results of the second type # 9 test in Table 3.).

To validate the stability and reliability of the novel method based on Poisson reconstruction degree fusion for detecting crack features at different depths in dynamic scenarios, the track maintenance robot system moves at a pre - set constant speed. It integrates an installed 2D optical camera and an infrared thermal imaging sensor to dynamically and real - time collect crack features. Subsequently, relevant experiments and data processing are conducted.A second set of samples, consisting of 9 groups of cracks with varying depths ranging from 0.35 to 8.00 mm, is selected for experimental testing. As illustrated in Figure 9 (a), this figure shows the relationship curve between crack feature points and feature grayscale values before fusion processing.During the dynamic acquisition of crack images, factors such as vibrations during the operation of the robot system lead to frame - dropping phenomena in 2D crack feature images. As a result, it becomes quite challenging to collect and recognize small - sized crack features. From Figure 9 (a), it is evident that there are very few feature pixels with grayscale values less than 60, and the majority of feature pixels have binary grayscale values in the range of 80 - 140. This distribution is mainly attributed to the dynamic real - time acquisition process.When performing dynamic real - time collection and identification of cracks, small - sized cracks pose relatively high recognition difficulties. Moreover, quantifying the geometric dimensions of cracks also presents certain challenges. Further fusion processing is then carried out, and the experimental results of crack testing under different Poisson reconstruction degrees are presented in Figures (b-j).

By examining Figures 9 (b - j), it is evident that the fused crack features are highly distinct. The gray values of the fused crack features reach the maximum Poisson reconstruction degree three times within the range of 20 - 80. Specifically, the Poisson reconstruction degree reaches its peak at both ends of the fused crack and also at the middle position of the crack during the dynamic collection and recognition process.Prior to the fusion using the Poisson reconstruction degree, while it was possible to detect the presence or absence of cracks to some extent, quantifying the crack size was challenging. However, after the fusion based on the Poisson reconstruction degree, the figure reveals that the features at both ends of the crack are accentuated. This enhancement greatly facilitates the quantitative analysis of the crack’s geometric dimensions.

**Fig.10:**
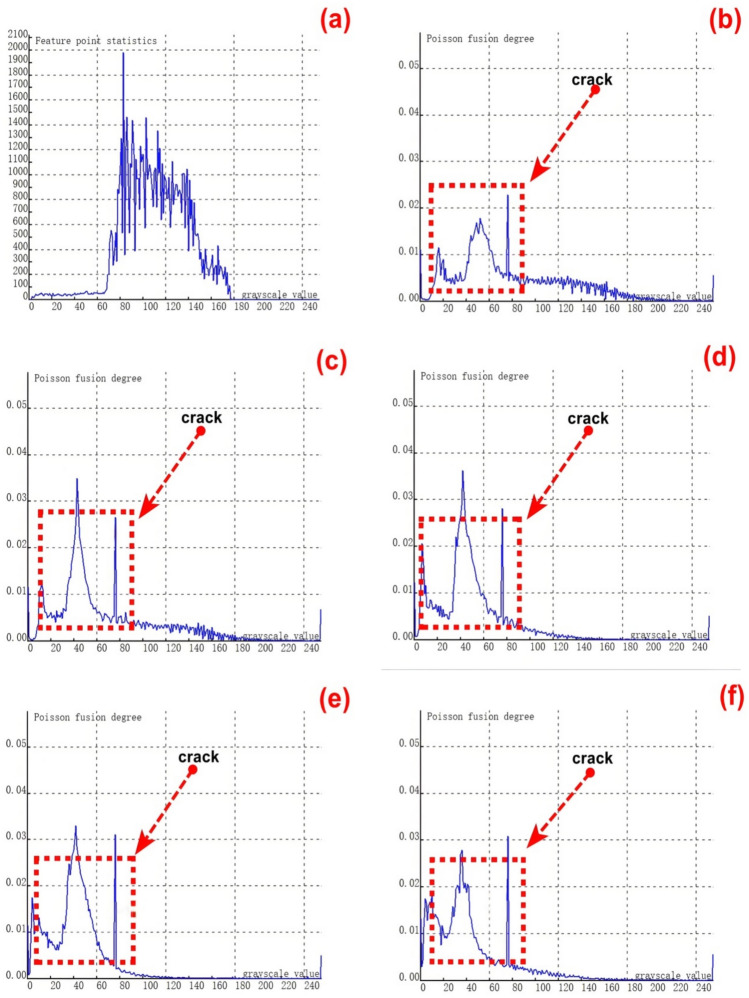

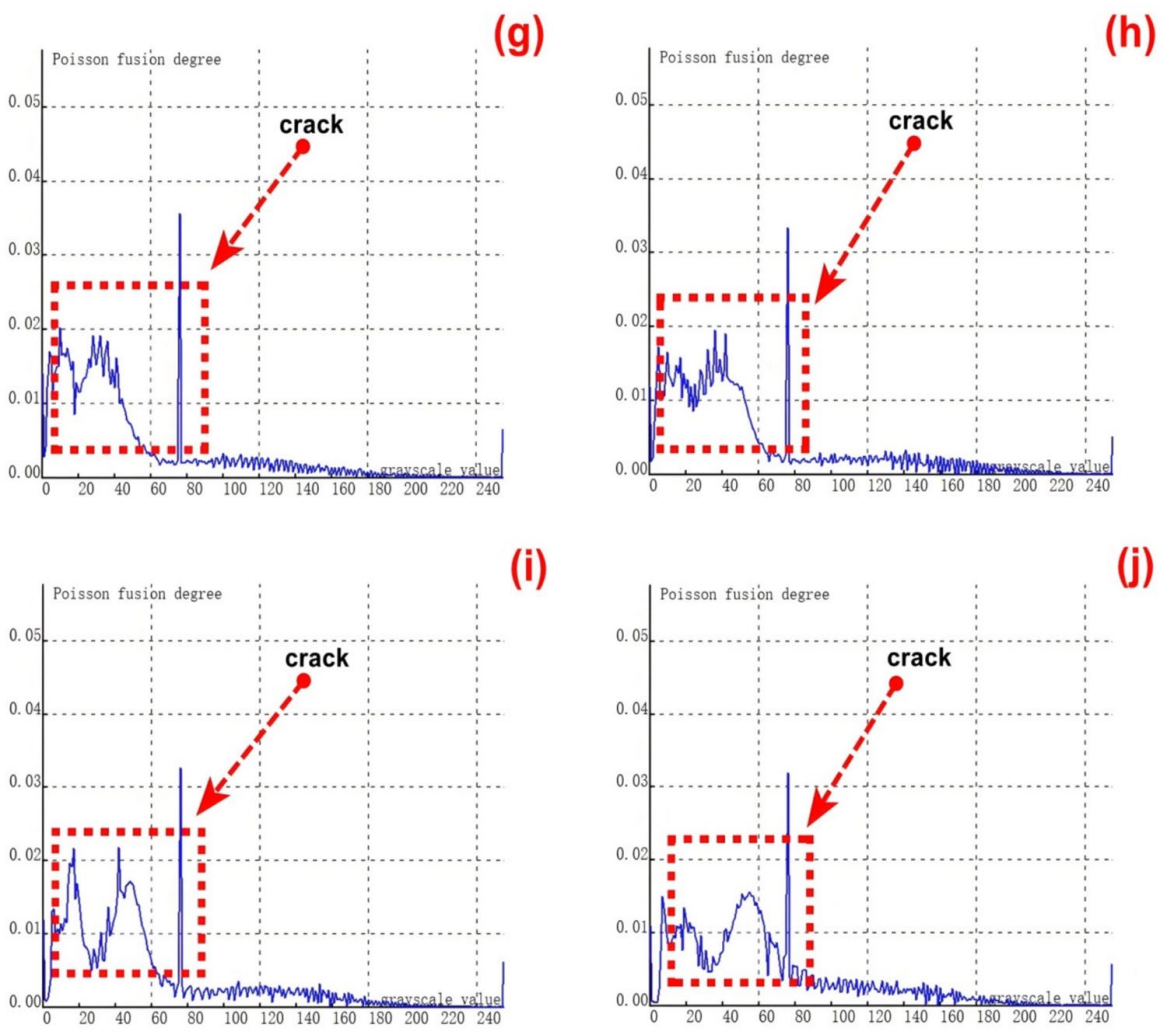
Quantitative depth and reference depth least squares fitting.

The relationship between the crack depth and the Poisson reconstruction degree of crack feature images was analyzed via least - squares fitting. As shown in Table 5 and Figure 10, there is an approximately monotonic and linear increase between the crack depth and the Poisson reconstruction degree.

**Table 5:** Quantitative results of crack testing based on Poisson reconstruction degree.

Reference crack depth(mm)	0.35	0.5	1.0	1.5	2.0	2.5	3.5	5	8
Maximum Poisson reconstruction degree	0.024	0.035	0.036	0.034	0.031	0.036	0.035	0.034	0.032
Quantitative depth(mm)	0.33	0.53	0.97	1.55	2.2	2.7	3.41	4.8	8.2
Quantitative error (%)	−0.06	0.06	−0.03	0.03	0.01	0.07	−0.03	−0.04	0.02
Mean Absolute Error	0.109								
Mean Squared Error	0.018

**Fig. 11:**
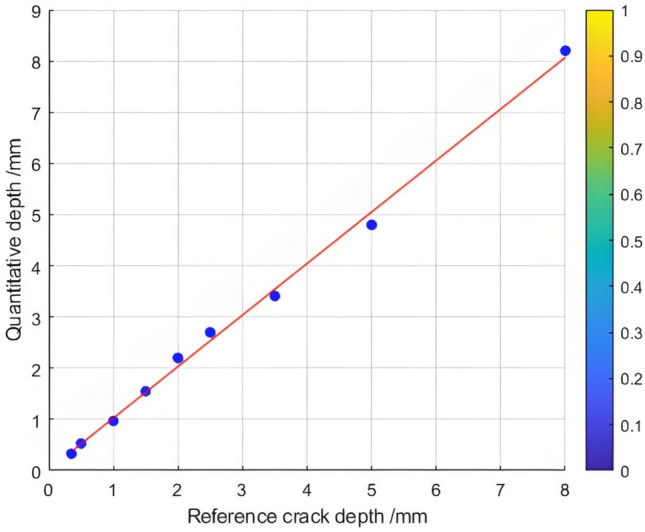
Quantitative depth and reference depth least squares fitting.

Consequently, the experimental data regarding crack depth were subjected to linear fitting. The quantification results are presented in Table 5 and Figure 11. The average absolute quantization error is 0.109%, and the mean - square quantization error is approximately 0.018%. These findings indicate that the Poisson reconstruction degree is effective in characterizing the crack depth under dynamic conditions.

Compared with the previous static test results, it can be found that the dynamic test results have high stability and small error. This is mainly because in the dynamic test process, the rail operation and maintenance robot moves back and forth on the rail at a constant speed, with the rolling friction between the wheel and the rail surface, resulting in a certain increase in the local temperature of the rail surface, which is superimposed on the electromagnetic induction heating temperature, and the temperature change is obvious. The damage characteristics of the infrared thermogram of the rail surface crack damage characteristics collected by the infrared thermography sensor are also more prominent. In the static test process, there is no temperature rise caused by the rolling friction between the wheel and the rail, so the infrared thermogram data collected in the dynamic state is more real and accurate than that in the static state. Especially in the real line detection, the high-speed rolling friction between the train wheel and the rail produces a higher temperature rise, so in the final result fusion analysis process,it is necessary to, The dynamic test results have less error than the static test results. At the same time, the dynamic test results are more consistent with the real-time online rail damage detection on the line.

### Experimental results of cracks of different lengths in different states

#### Experimental test results of different crack lengths under dynamic conditions

**Fig. 12:**
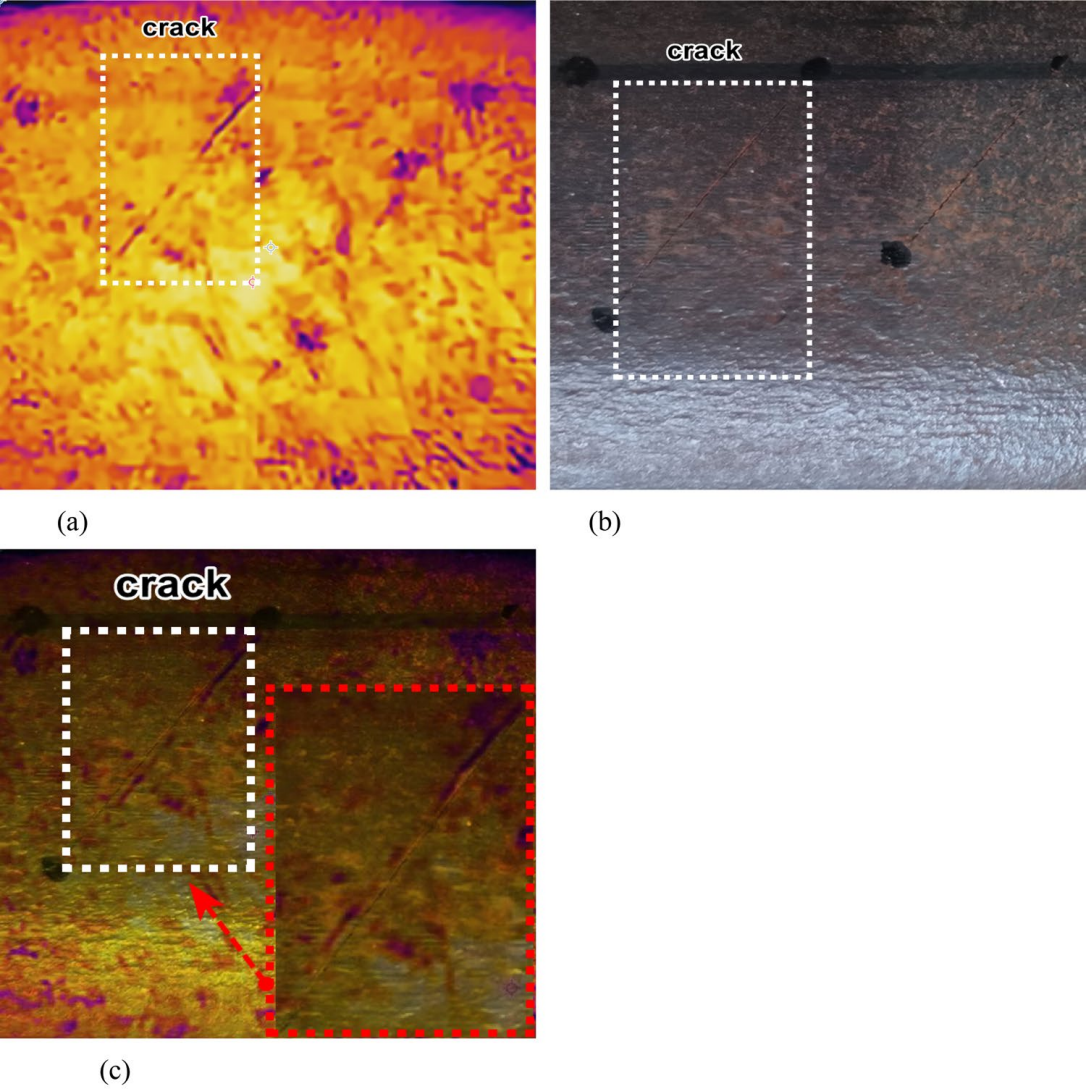
Fusion processing of infrared thermal images of cracks.

A first set of samples, consisting of 9 groups of cracks with different lengths ranging from 1 to 10.00 mm, was selected for experimental testing. As depicted in Figure 12, it presents the feature fusion processing diagram for a crack with a length of 5 mm, which is the intermediate value among the selected crack lengths.Figure 12 (b) showcases the feature display of cracks in 2D optical images. From this figure, it is evident that only some of the crack features are emphasized, and it is not possible to comprehensively and clearly capture the entire crack features. This is because, during the dynamic acquisition process, there are certain frame - dropping issues caused by external factors.Figure 12 (a) illustrates the feature display of cracks in infrared thermal images, while Figure 12 (c) shows the fused crack feature map.

**Fig. 13:**
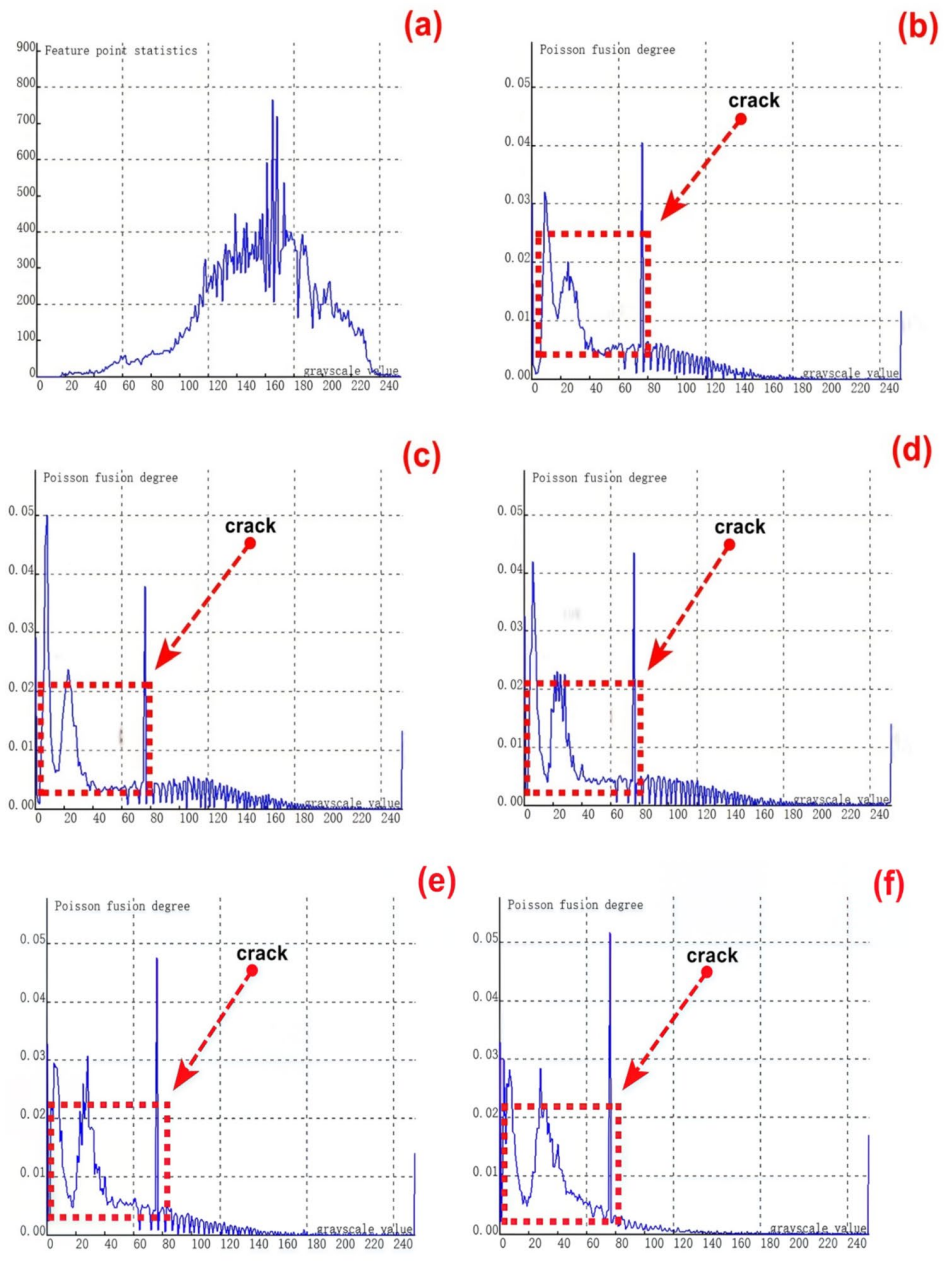

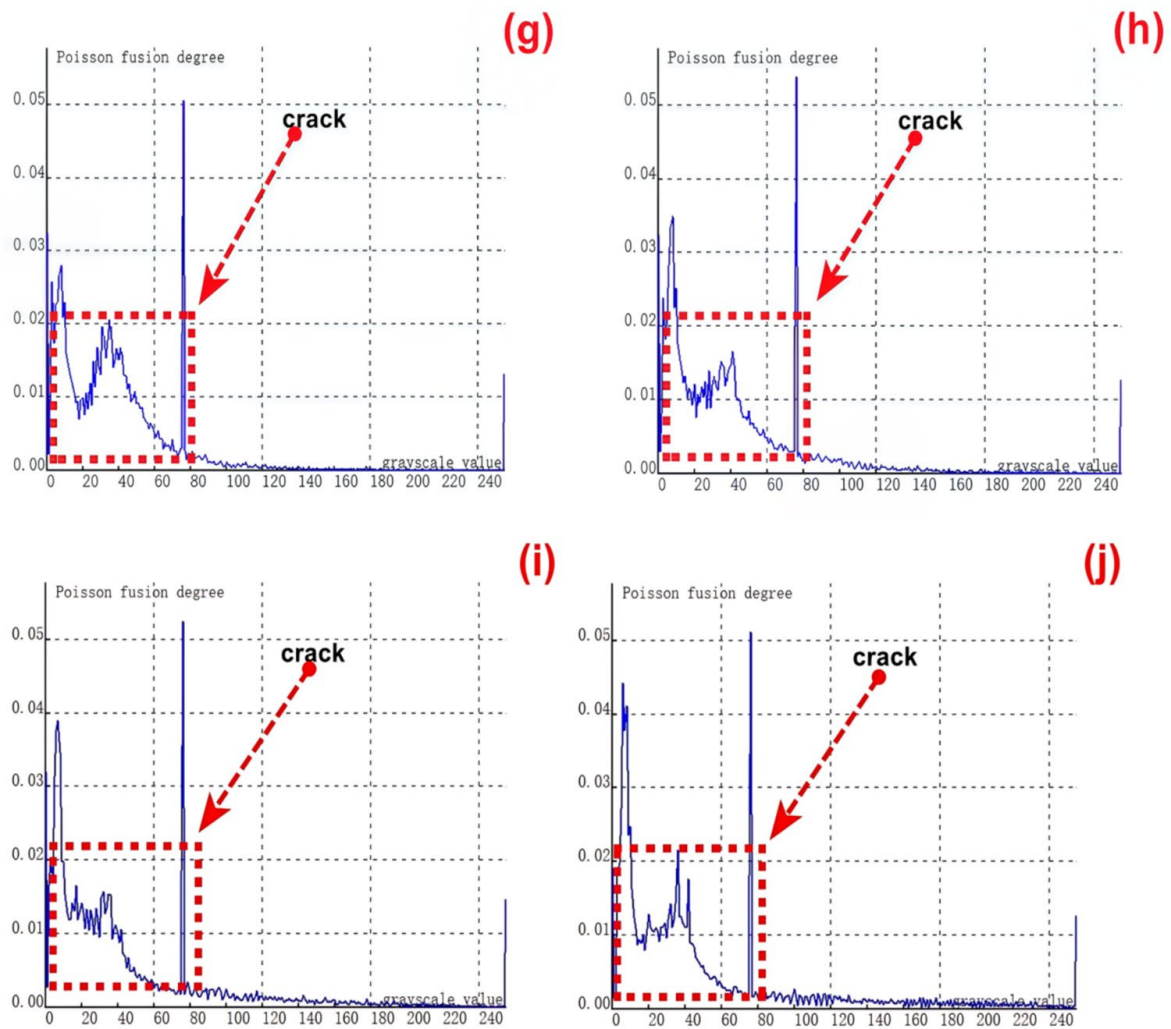
Poisson reconstruction degree relationship curve of cracks of different lengths under dynamic conditions. ((**a**). Control group; (**c**). Analysis results of the first type # 2 test in Table 3; (**d**). Analysis results of the first type # 3 test in Table 3; (**e**). Analysis results of the first type # 4 test in Table 3; (**f**). Analysis results of the first type # 5 test in Table 3; (**g**). Analysis results of the first type # 6 test in Table 3; (**h**). Analysis results of the first type # 7 test in Table 3; (**i**). Analysis results of the first type # 8 test in Table 3; (**j**). Analysis results of the first type # 9 test in Table 3.).

As depicted in Figure 13, experimental tests were carried out on the first - category cracks, which consist of 9 groups of cracks with varying lengths. Under dynamic conditions, the sum of feature pixels at both ends and the middle position of cracks of different lengths was computed. Subsequently, this sum was compared with the corresponding values obtained after fusion based on the Poisson fusion degree.

Figure 13 (a) presents the crack feature map prior to fusion, while Figures (b - j) display the experimental results of crack testing under different Poisson reconstruction degrees.By examining Figures 13 (b - j), it becomes apparent that the fused crack features are highly distinct. Within the Poisson reconstruction degree range of 10 - 70, the gray values of the fused crack features reach their maxima twice. Moreover, the maximum values from the earlier instance are lower than those from the second instance.The data indicates that during the dynamic real - time acquisition and recognition of crack features, the crack image acquisition process is influenced by a certain speed due to the impact of the acquisition frame rate. As a result, the gray values of the crack features after the first fusion are smaller compared to those after the second fusion. This phenomenon accentuates the features at both ends of the crack during dynamic identification, thereby facilitating the quantitative analysis of the crack’s geometric dimensions.

**Fig.14:**
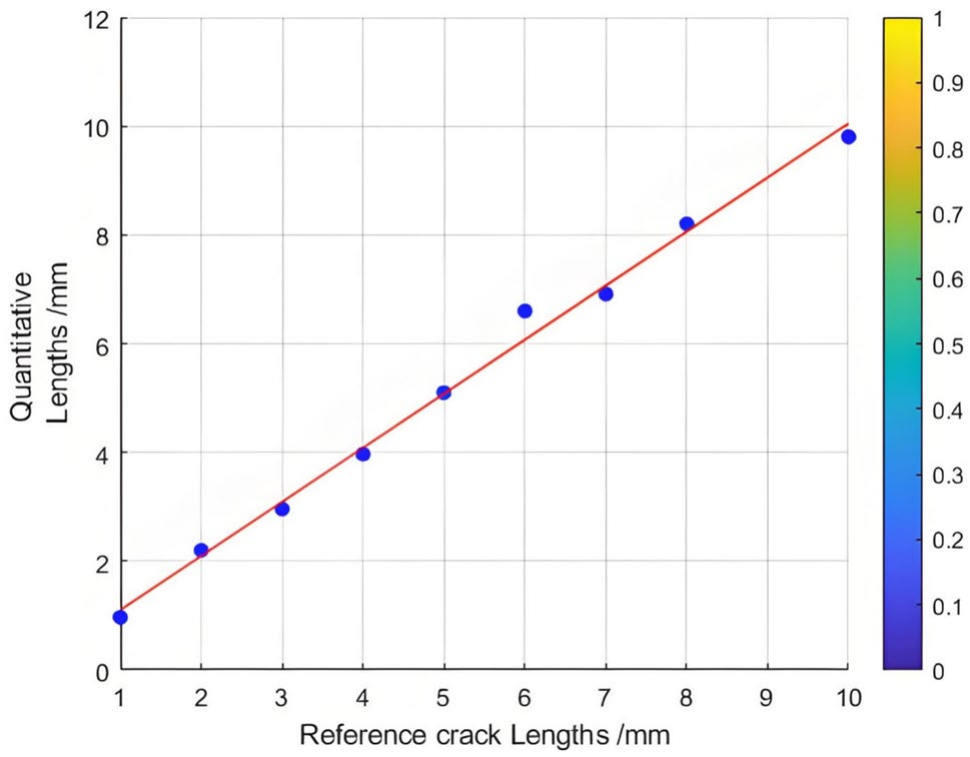
Quantitative length and reference length least squares fitting.

The relationship between the crack length and the Poisson reconstruction degree of crack feature images was analyzed using the least - squares method. As presented in Table 6, there is an approximately monotonic and linear increase between the crack length and the Poisson reconstruction degree.Consequently, the experimental data on crack length were used for linear fitting. The quantification results are shown in Table 6 and Figure 14. The average absolute quantization error is 0.175%, and the mean - square quantization error is approximately 0.049%. These results indicate that the Poisson reconstruction degree is effective in characterizing the crack length under dynamic conditions.Table 6 also presents the quantitative results of testing cracks with different lengths under dynamic states.

**Table 6:** Quantitative results of crack testing based on Poisson fusion degree.

Reference crack lengths (mm)	1	2	3	4	5	6	7	8	10
Maximum poisson fusion degree	0.040	0.038	0.043	0.047	0.052	0.050	0.053	0.053	0.052
Quantitative lengths (mm)	0.97	2.2	2.96	3.97	5.1	6.6	6.91	8.2	9.8
Quantitative error (%)	−0.03	0.09	−0.01	−0.04	0.01	0.09	−0.13	0.02	−0.02
Mean absolute error	0.175								
Mean squared error	0.049

#### Experimental test results of different crack lengths under static conditions

**Fig. 15:**
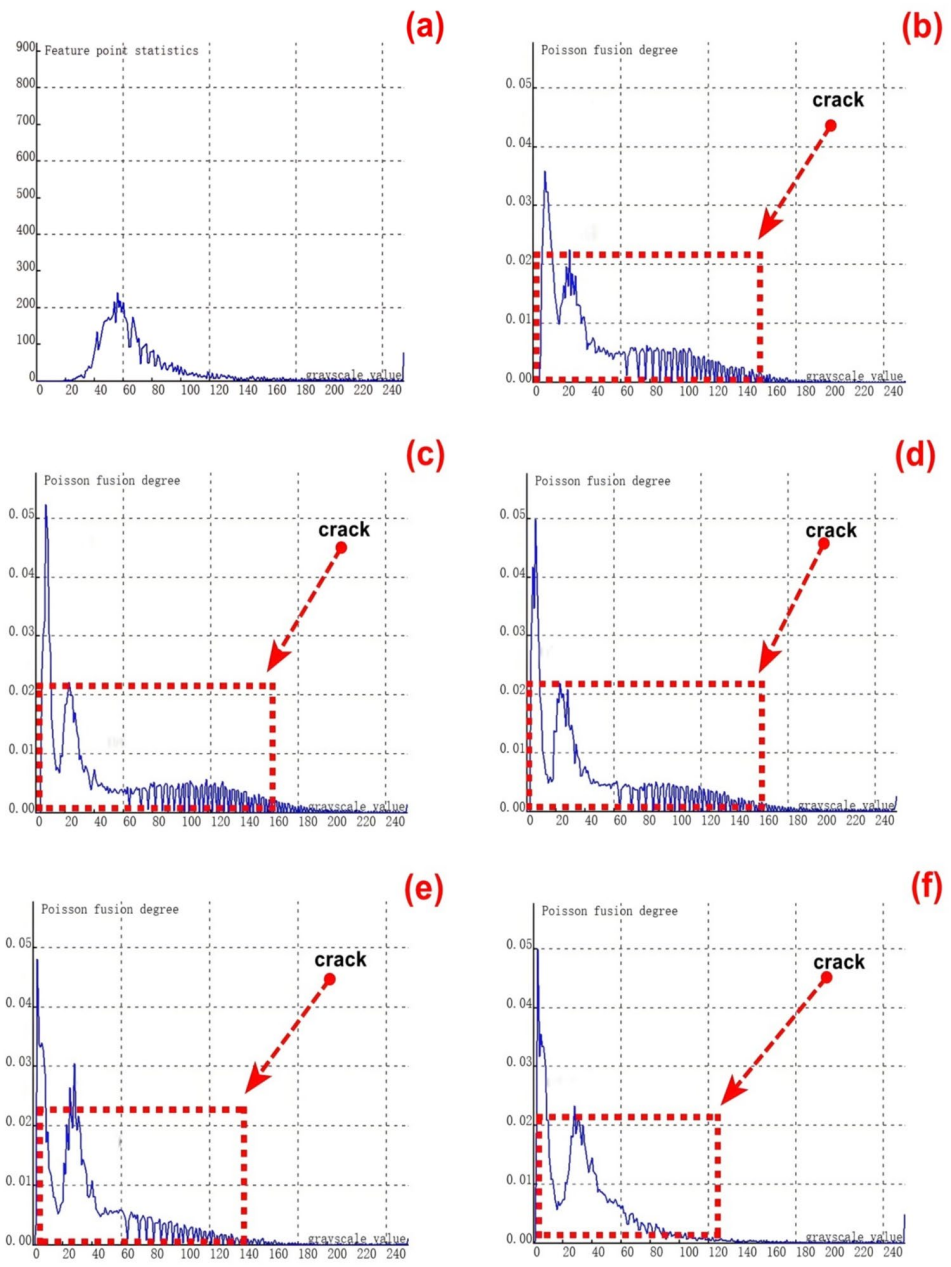

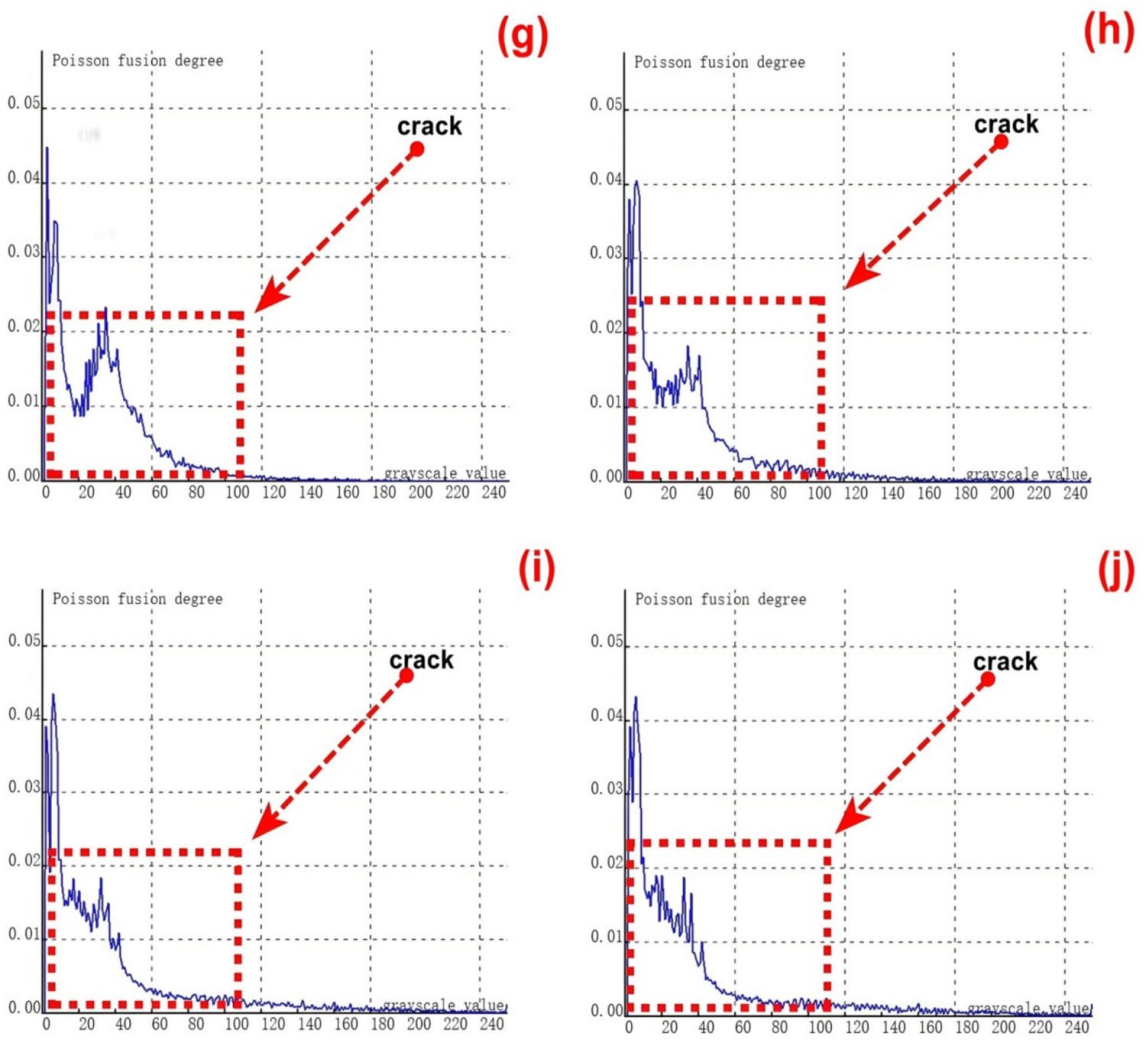
Poisson reconstruction degree relationship curve of cracks of different lengths under static conditions. ((**a**). Control group; (**c**). Analysis results of the first type # 2 test in Table 3; (**d**). Analysis results of the first type # 3 test in Table 3; (**e**). Analysis results of the first type # 4 test in Table 3; (**f**). Analysis results of the first type # 5 test in Table 3; (**g**). Analysis results of the first type # 6 test in Table 3; (**h**). Analysis results of the first type # 7 test in Table 3; (**i**). Analysis results of the first type # 8 test in Table 3; (**j**). Analysis results of the first type # 9 test in Table 3.).

To validate the stability and reliability of the novel method for feature - fusion detection of cracks with different lengths, which is based on Poisson reconstruction degree fusion, experimental tests were carried out under static conditions.A first set of samples, consisting of 9 groups of cracks with lengths varying from 1 to 10.00 mm, was selected for these tests. The results of the experimental tests are presented in Figures 15 (a - j).

As depicted in Figure 15 (a), the crack feature map without Poisson reconstruction degree fusion reveals that the number of feature pixels reaches its peak when the grayscale value is 50. This phenomenon occurs because, during the recognition of static crack features, the feature in the middle of the crack is the most prominent and distinct. Within the entire grayscale value range of 30 - 100 for crack features, there are relatively fewer feature pixels at both ends of the crack. This accounts for the difficulty in identifying and detecting some extremely small and slender cracks under static conditions.Figures (b-j) illustrate the crack feature curves after Poisson reconstruction degree fusion. It is evident from these figures that the grayscale value range of crack features spans from 10 to 140. After the fusion process, the features at both ends of the crack become more pronounced compared to the pre - fusion state. Consequently, there are relatively larger Poisson reconstruction degree values at grayscale values of 10 and in the range of 100 - 140, respectively.

**Fig. 16:**
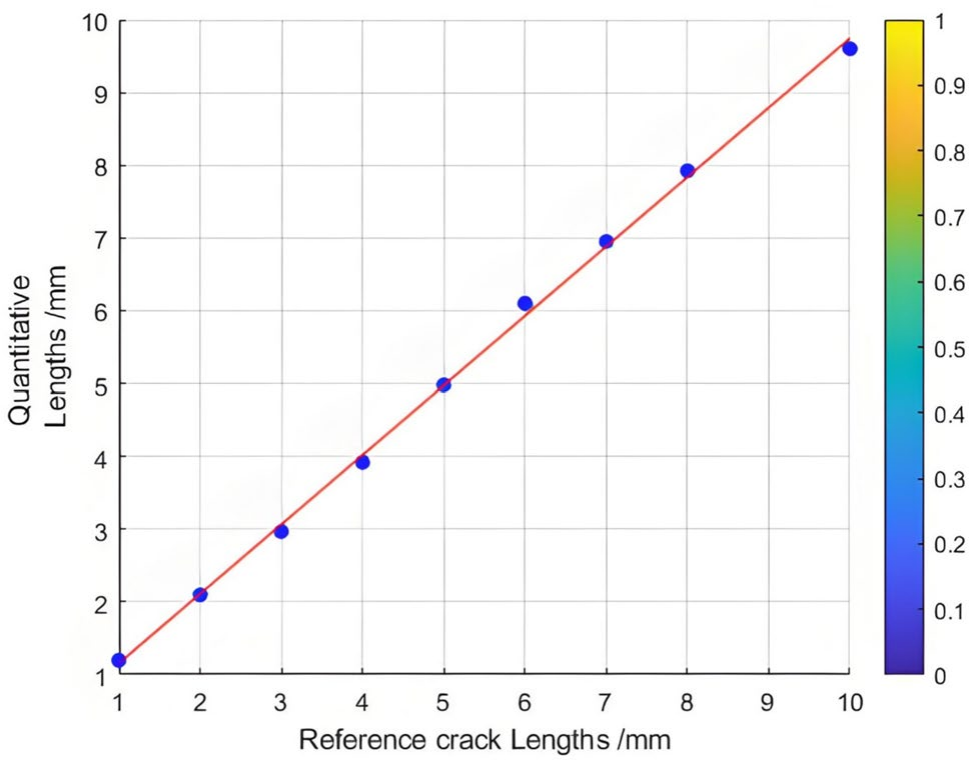
Quantitative length and reference length least squares fitting.

The relationship between the crack length and the Poisson reconstruction degree of crack feature images was analyzed using the least - squares method. As shown in Table 7, there is an approximately monotonic and linear decrease between the crack length and the Poisson reconstruction degree.Consequently, the experimental data of crack length were subjected to linear fitting. The quantification results are presented in Table 7 and Figure 16. The average absolute quantization error stands at 0.08%, and the mean - square quantization error is approximately 0.009%. These results indicate that the Poisson reconstruction degree is effective in characterizing the crack length under static conditions.Table 7 also shows the quantitative results of testing cracks with different lengths under static states.

**Table 7:** Quantitative results of crack testing based on Poisson fusion degree.

Reference crack lengths (mm)	1	2	3	4	5	6	7	8	10
Maximum poisson fusion degree	0.036	0.053	0.050	0.048	0.049	0.046	0.040	0.043	0.042
Quantitative lengths (mm)	1.2	2.1	2.97	3.92	4.98	6.1	6.95	7.92	9.6
Quantitative error (%)	0.17	0.05	−0.01	−0.02	−0.01	0.02	−0.01	−0.01	−0.04
Mean absolute error	0.080								
Mean squared error	0.009

### Comparative experimental results of artificial cracks and natural RCF cracks

This section conducts comparative experimental testing and analysis between artificially manufactured cracks and natural RCF cracks, further demonstrating the effectiveness of Poisson’s fusion degree in identifying crack damage characteristics.

#### Experimental test results of different types of artificial cracks

**Fig. 17:**
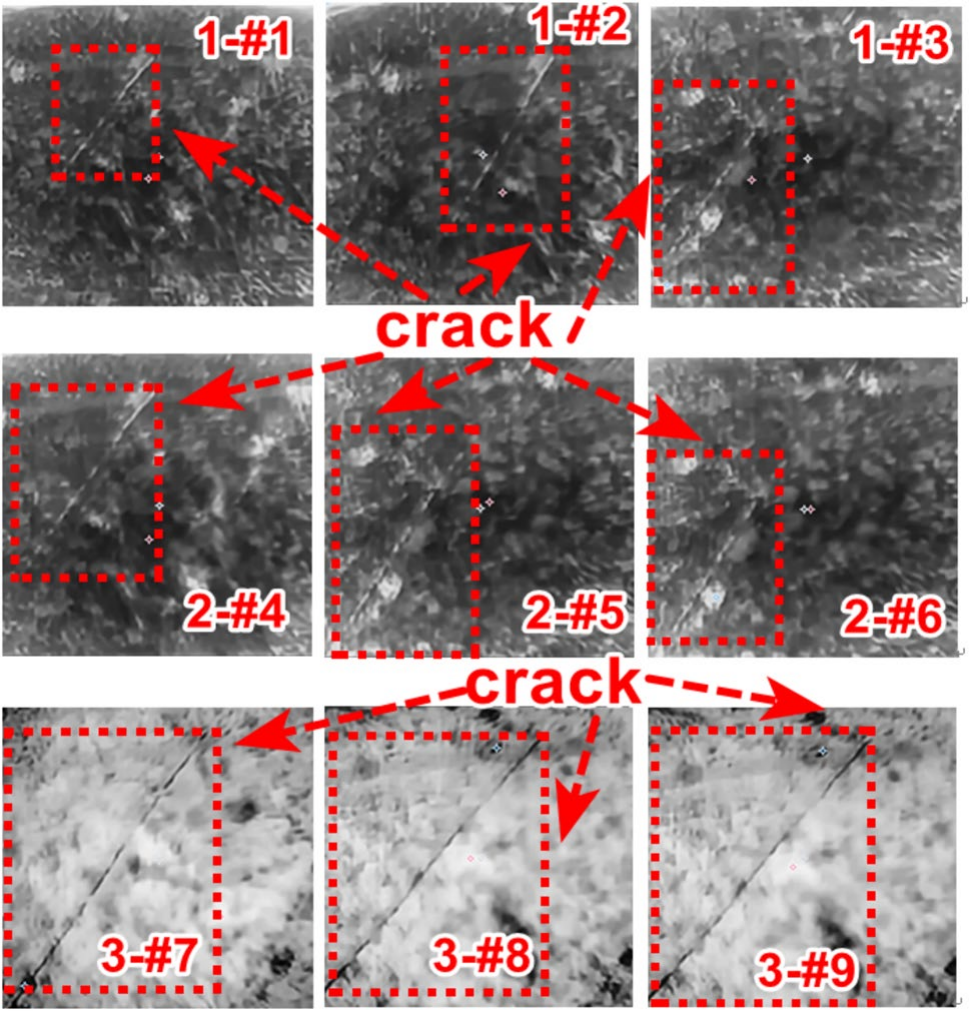
Thermal map of different types of crack features after fusion.

For experimental testing, three sets of crack types were selected. The first set includes crack specimens labeled as #1, #2, and #3; the second set comprises specimens #4, #5, and #6; and the third set consists of specimens #7, #8, and #9. The corresponding crack lengths span from 1 mm to 10 mm, while the crack depths range from 1.5 mm to 8 mm.Figure 17 presents the crack feature map after the application of Poisson reconstruction degree fusion. In this figure, the notation “1 - #1” designates the first crack specimen of the first type, as detailed in Table 1. The same naming convention applies to the other specimens accordingly.

To verify the credibility of the proposed method, three different types of artificial cracks will be selected and tested in the same way as the high-speed railway track rails that will be compared later. The analysis of the test results for artificial cracks is shown in Table 7.

**Fig. 18:**
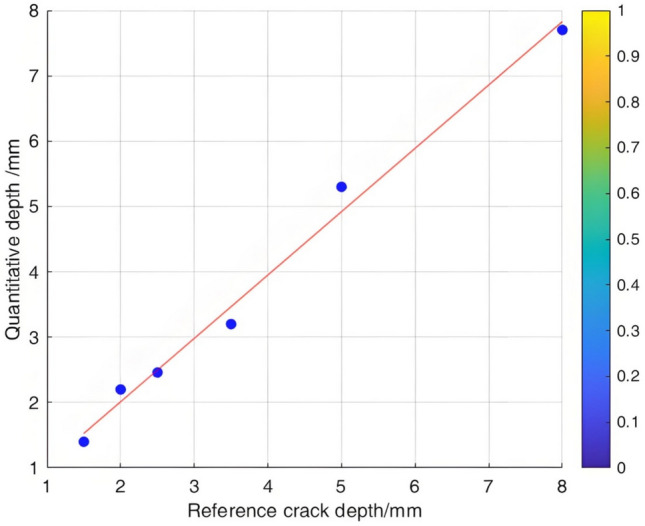
Quantitative length and reference length least squares fitting.

A comparative analysis was conducted on crack lengths of 1 mm, 2 mm, 3 mm, 7 mm, 8 mm, and 10 mm. The results reveal that for these crack lengths, the crack features become more prominent, with the middle - part features being more distinct. This indicates that the fusion process significantly enhances the identifiability of crack features.The corresponding calculated values of the Poisson fusion degree are presented in Table 7. To further understand the relationship between the crack length and the Poisson reconstruction degree of crack feature images, the least - squares method was employed for fitting and analysis. As shown in Table 8, there is an approximately monotonic and linear decrease between the crack length and the Poisson reconstruction degree.Based on these findings, the experimental data of crack length were subjected to linear fitting. The quantification results are depicted in Figure 18. The average absolute quantification error is 0.198%, and the quantification mean - square error is approximately 0.043%. These results suggest that the Poisson reconstruction degree is effective in characterizing crack lengths under different conditions.

**Table 8:** Quantitative results of different types of crack testing based on poisson fusion degree.

Reference crack lengths (mm)	1	2	3	7	8	10
Maximum poisson fusion degree	0.035	0.055	0.052	0.045	0.046	0.043
Quantitative lengths (mm)	1.3	1.95	2.98	7.2	8.3	9.7
Quantitative error (%)	0.23	−0.03	−0.01	0.03	0.04	−0.03
Mean absolute error	0.198					
Mean squared error	0.043

**Fig. 19:**
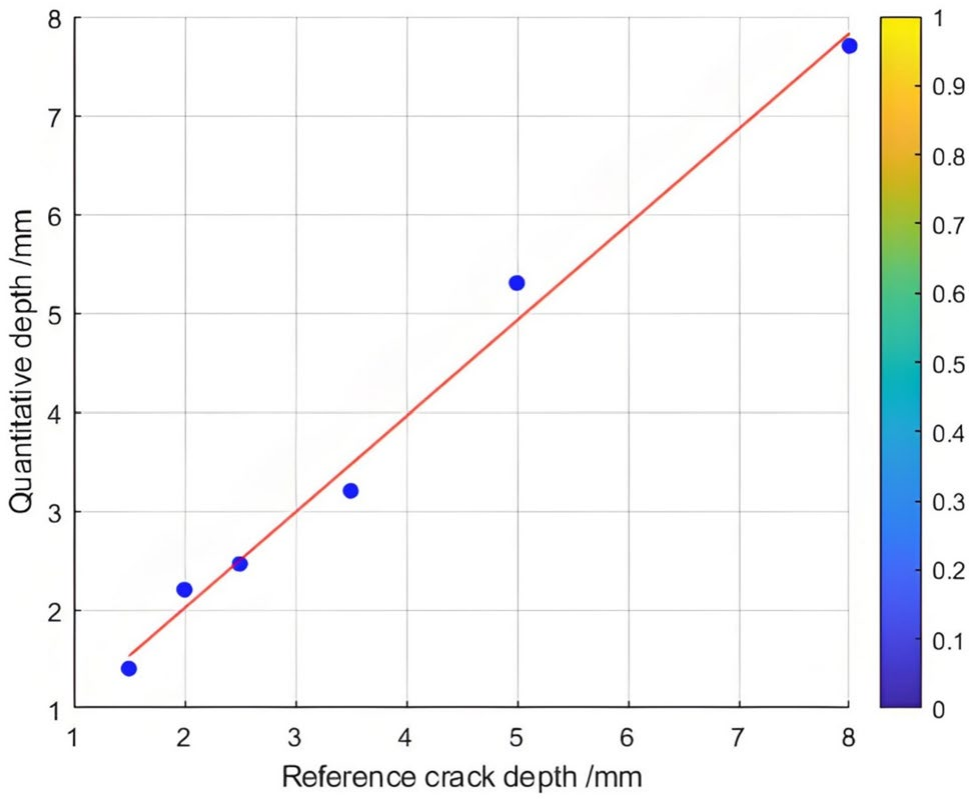
Quantitative depth and reference depth fitting using least squares method.

Similarly, a comparative analysis was performed on crack depths spanning from 1.5 mm, 2.0 mm, 2.5 mm, 3.5 mm, 5 mm, to 8 mm. The corresponding calculated values of the Poisson reconstruction degree are presented in Table 9.To explore the relationship between the crack depth and the Poisson reconstruction degree of crack feature images, the least - squares method was employed for fitting and analysis. As indicated in Table 9, there is an approximately monotonic decrease between the crack depth and the Poisson reconstruction degree.Consequently, the experimental data on crack depth were utilized for linear fitting. The quantitative results are depicted in Figure 19. The average absolute quantification error is 0.188%, and the quantification mean - square error is approximately 0.047%. These results suggest that the Poisson reconstruction degree effectively characterizes crack depths under various conditions.

**Table 9:** Quantitative results of different types of crack testing based on poisson fusion degree.

Reference crack depth (mm)	1.5	2	2.5	3.5	5	8
Maximum poisson fusion degree	0.035	0.046	0.052	0.053	0.056	0.054
Quantitative depth (mm)	1.4	2.2	2.46	3.2	5.3	7.7
Quantitative error (%)	−0.07	0.09	−0.03	−0.09	0.06	−0.04
Mean absolute error	0.188					
Mean squared error	0.047

#### Natural RCF crack experimental test results

**Fig. 20:**
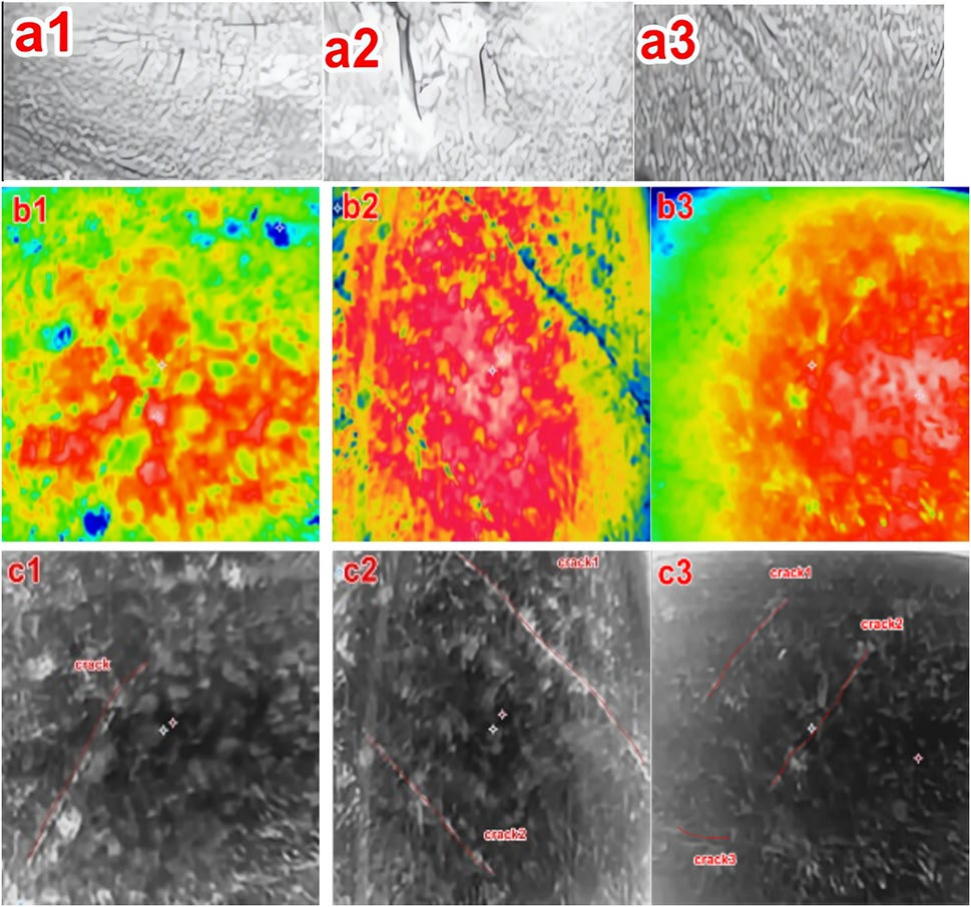
Experimental sample crack.

To further validate the stability and reliability of the proposed Poisson reconstruction degree, natural cracks in high - speed railway tracks were employed for experimental testing. Three experimental specimens were cut from 60 - kg steel rails used in high - speed railway tracks, as illustrated in Figures 20 (a1 - a3).The majority of rolling contact fatigue (RCF) cracks in these specimens have extremely small depths and lengths, on the order of a few hundred micrometers. Therefore, static crack feature collection and testing methods were adopted. Figures 20 (b1 - b3) present the infrared thermograms of the crack characteristics of the experimental specimens before the fusion process. These thermograms reveal that it is challenging to detect natural RCF cracks during the fusion treatment, as the crack characteristics are not particularly distinct.In contrast, Figures 20 (c1 - c3) show the crack feature maps after fusion based on the Poisson reconstruction degree. It is evident that the crack features at this stage are more prominent compared to those in Figures 20 (b1 - b3), facilitating their identification.

**Fig. 21:**
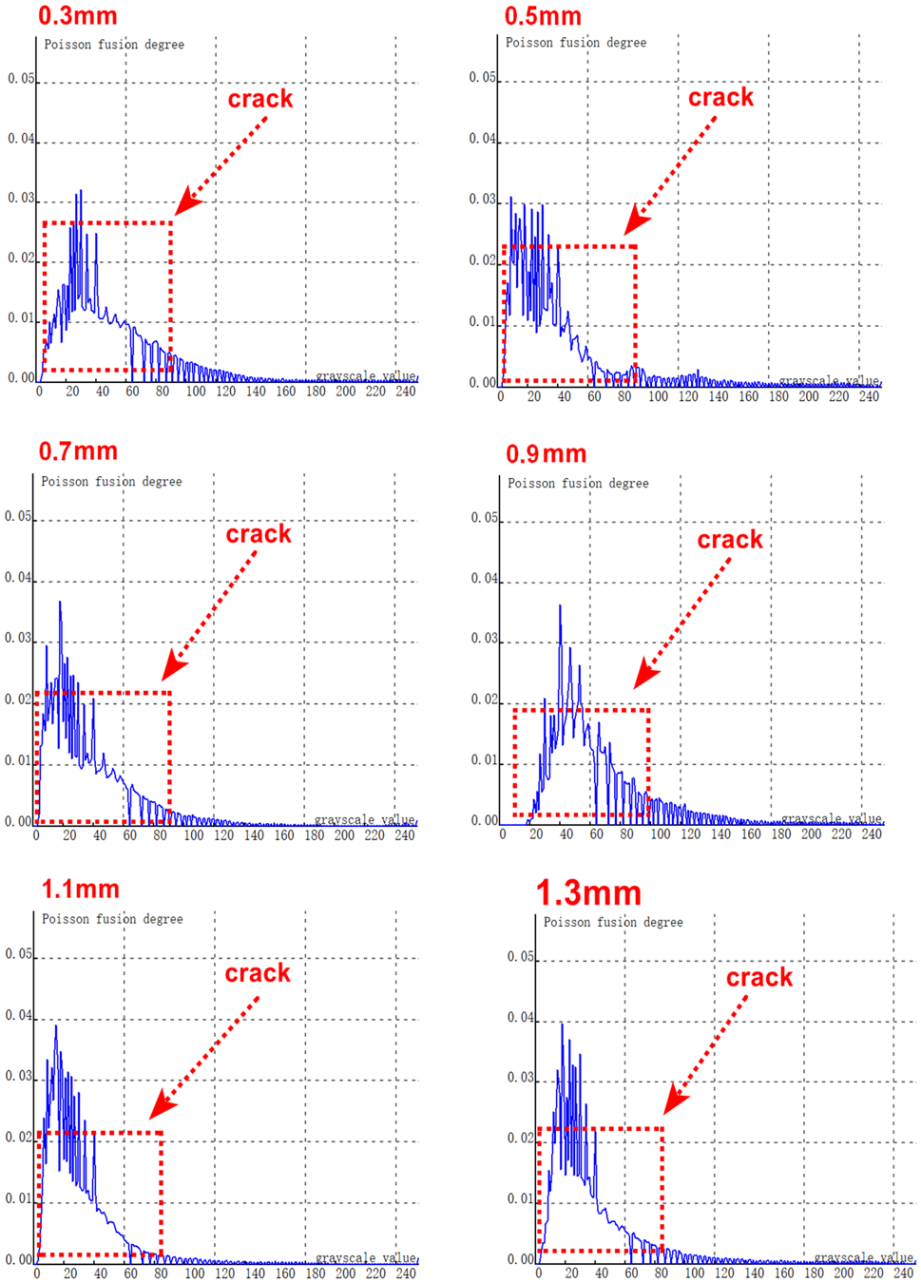
Relationship curve between natural RCF cracks and Poisson reconstruction degree.(0.3mm natural real crack test results; 0.5mm natural real crack test results; 0.7mm natural real crack test results; 0.9mm natural real crack test results; 1.1mm natural real crack test results; 1.3mm Natural True Crack Test Results).

**Fig. 22:**
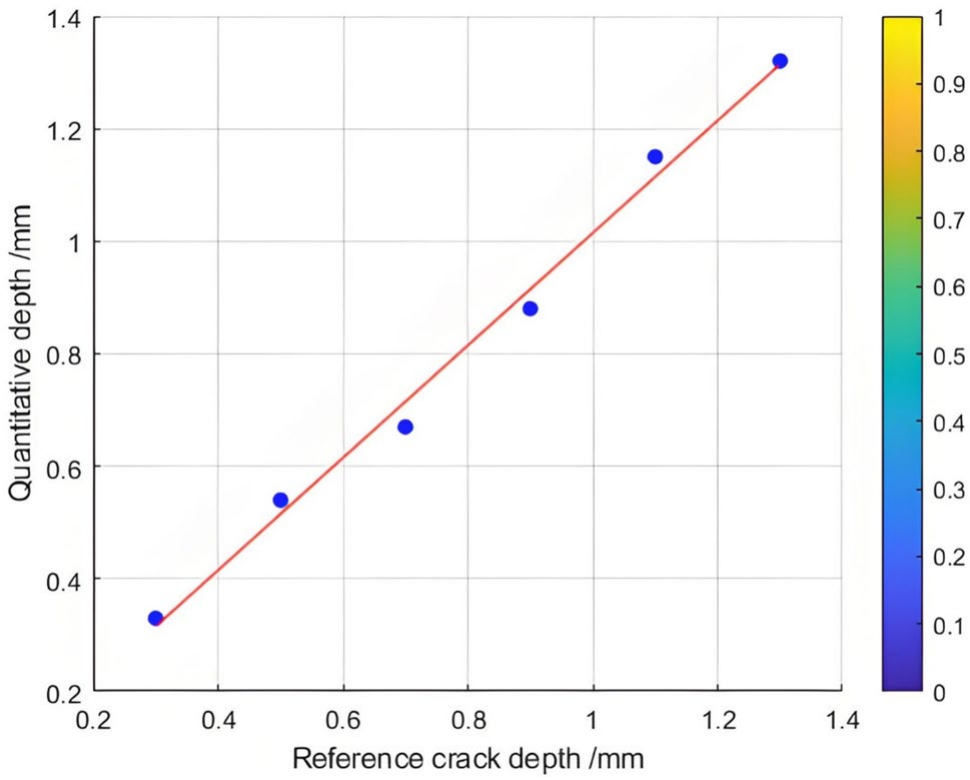
Quantitative depth and reference depth least squares fitting.

The depth of the selected natural rolling contact fatigue (RCF) cracks for cutting ranges from 0.3 mm to 1.5 mm. Several cracks were chosen for testing, and their characteristics were statistically confirmed through industrial computed tomography (CT). Figure 21 depicts the relationship curve between the feature pixels of natural RCF cracks and the Poisson reconstruction degree.As can be observed from the figure, akin to the test results of the previous artificial experimental samples, after the fusion process, the Poisson reconstruction degree reaches its maximum value twice within the range of 10 - 80 and also exhibits a linear proportional relationship. The measurement results of natural RCF cracks based on the Poisson reconstruction degree are largely consistent with those of artificial cracks. The specific quantitative results are presented in Table 10.To further quantify the relationship between the depth of natural RCF cracks and the Poisson reconstruction degree, the least - squares method was employed for comparison and analysis of the results. The quantitative results, analyzed using the least - squares approach, yield an average absolute quantization error of 0.027% and a mean - square quantization error of approximately 0.03%, as shown in Figures 21 and 22. These findings indicate that the Poisson reconstruction degree is effective in characterizing the crack depth of natural RCF cracks.

**Table 10:** Quantitative results of different types of crack testing based on poisson fusion degree.

Reference crack depth (mm)	0.3	0.5	0.7	0.9	1.1	1.3
Maximum poisson fusion degree	0.032	0.032	0.036	0.0 37	0.038	0.040
Quantitative depth (mm)	0.33	0.54	0.67	0.88	1.15	1.32
Quantitative error (%)	0.143	0.074	−0.045	−0.023	0.043	0.015
Mean absolute error	0.027					
Mean squared error	0.030

## Conclusion and future work

This paper presents a method of fusing 2D optical images and eddy - current thermal images based on the Poisson reconstruction degree to quantitatively determine the length and depth of rail cracks under both static and dynamic detection scenarios. By statistically analyzing the Poisson reconstruction degree, a relationship between the physical mechanism of pulse heating imaging and 2D optical image fusion was established. The quantitative accuracy of rail crack measurements based on the Poisson reconstruction degree was compared and evaluated in static and dynamic detection modes. The following conclusions can be drawn:

1. In the static detection mode, the proposed Poisson reconstruction degree model exhibits a monotonic relationship with the crack length and depth. Moreover, the quantification accuracy of crack length and depth in the static mode is higher than that in the dynamic mode, as evidenced by the smaller quantization errors for length and depth in the static mode compared to the dynamic mode.
2. After calibrating the corresponding regression coefficients and correction relationships for different types of crack damage in both static and dynamic modes, the accuracy of quantitatively measuring crack length and depth using the Poisson reconstruction degree is independent of the crack type and shows a linear relationship.
3. During the testing of natural rolling contact fatigue (RCF) cracks, the quantitative analysis based on the Poisson reconstruction degree reveals a linear relationship between the length and depth of natural RCF cracks. This indicates that the Poisson reconstruction degree is effective in characterizing the crack depth of natural RCF cracks.

Considering the real and actual working conditions of the track rails on the railway line, as well as the surface crack damage of the rails on the line, the artificial manufacturing of cracks in the text is carried out according to the standard "Railway Line Repair Rules" (TieYun [2006] No. 146), aiming to achieve the characteristics of rail crack damage that are close to reality. At the same time, multiple experimental tests are conducted in a cross mixed state to further improve the credibility and feasibility of the proposed method. In the final testing experiment, the rail crack damage on the real track was selected according to the standard, and multiple comparative experiments were conducted with artificially manufactured crack damage. The test results were analyzed to further verify the feasibility of the method.

The key direction of future research work will be to utilize more AI algorithms to further improve the accuracy of the damage detection method proposed in the article. The proposed method, equipped with a self-developed track operation and maintenance robot system, will be jointly tested with Guangdong CRRC to conduct online real-time detection on real lines. After the test is completed, if the test results are satisfactory, the expected results will be achieved. Next, we will conduct research and development on AI based damage detection algorithms to achieve intelligent and digital online real-time rail damage detection.

## Data Availability

The datasets generated and/or analysed during the current study are not publicly available due [This research project is a major ongoing scientific research project, which involves key enterprises in the relevant industry and is protected by technical confidentiality agreements signed with partner enterprises. It is precisely these agreements that restrict the public sharing of data to safeguard the intellectual property rights and business interests of the partnering enterprises. We have obtained all the necessary permissions for using these data within the scope of this thesis publication. However, unfortunately, we are unable to make these data publicly available.] but are available from the corresponding author on reasonable request.
